# Whole-Language and Item-Specific Inhibition in Bilingual Language Switching: The Role of Domain–General Inhibitory Control

**DOI:** 10.3390/brainsci10080517

**Published:** 2020-08-05

**Authors:** Judy D. Zhu, Paul F. Sowman

**Affiliations:** 1 Department of Cognitive Science, Macquarie University, Sydney 2109, Australia; paul.sowman@mq.edu.au; 2 ARC Centre of Excellence in Cognition and its Disorders, Sydney 2109, Australia

**Keywords:** bilingualism, language control, whole-language inhibition, item-specific inhibition, TMS, pre-SMA

## Abstract

A prominent theory of bilingual speech production holds that appropriate language selection is achieved via inhibitory control. Such inhibition may operate on the whole-language and/or item-specific level. In this study, we examined these two levels of control in parallel, by introducing a novel element into the traditional cued language switching paradigm: half of the stimuli were univalent (each required naming in the same language every time it appeared), and the other half were bivalent (each required naming in different languages on different trials). Contrasting switch and stay trials provided an index for whole-language inhibition, while contrasting bivalent and univalent stimuli provided an index for item-specific inhibition. We then investigated the involvement of domain-general brain mechanisms in these two levels of language control. Neuroimaging studies report activation of the pre-supplementary motor area (pre-SMA), a key region in the executive control brain network, during language switching tasks. However, it is unclear whether or not the pre-SMA plays a causal role in language control, and at which level it exerts control. Using repetitive transcranial magnetic stimulation (TMS) to transiently disrupt the pre-SMA, we observed an essential role of this brain region in general speech execution, while evidence for its specific involvement in each level of inhibition remains inconclusive.

## 1. Introduction

At least half of the world’s population today is bilingual or multilingual [1]. Knowing more than one language comes with the benefit of having access to information from a wider range of sources, as well as being able to communicate with more diverse groups of people. At the same time, it demands some kind of control mechanism to keep the languages separate and to ensure they do not interfere with each other. How do bilingual individuals coordinate their two languages successfully, so that they can speak the desired language at any given time? How do they switch between languages with ease? An influential view was put forward by Green [2], in his “inhibitory control model” of bilingual speech production. According to this model, appropriate language control is achieved via inhibition of the non-target language. That is, when bilinguals speak one language, they need to suppress the other language to avoid interference. Based on the assumption that lexical items in the more dominant language have a higher level of baseline activation, Green proposes that stronger inhibition needs to be placed on that language in order to enable speech production in the non-dominant language. On the other hand, production in the dominant language does not require that as much inhibition be applied to the non-dominant language. This leads to the prediction that it is relatively more difficult for bilinguals to return to their dominant language after speaking in their non-dominant language (than the other way around), due to the need to overcome stronger prior inhibition.

### 1.1. Behavioural Markers of Inhibition

Green’s inhibition account of bilingual control [2] finds support in the cued language switching paradigm [3,4,5,6,7,8]. In this paradigm, bilingual participants name pictures or numerals in their first language (L1) or second language (L2), according to a cue given on each trial. The language requirement can either change from the previous trial (*switch trial*) or stay the same as the previous trial (*stay trial*). A robust finding is that reaction times (RT) are longer on switch trials compared to stay trials. This RT difference is commonly referred to as the *switch cost*. While the switch cost itself can be attributed to a number of factors, such as cue encoding [9], task shifting, and goal updating [10], it is the finding of asymmetrical switch cost (especially in unbalanced bilinguals) that points towards the involvement of inhibitory processes in language switching.

*Asymmetrical switch cost* refers to the observation that the switch cost is larger when bilinguals switch into the dominant language, compared to switching into the non-dominant language [3,4,6,7,8,11,12,13]. This pattern aligns with Green’s inhibition account of language control: production in the non-dominant language requires stronger suppression of the dominant language, so it takes more time to overcome such suppression when switching back into the dominant language [2]. This phenomenon is replicated in many studies (see above), but it is not universally observed. For example, the switch cost asymmetry seems to disappear when participants are given long preparation times for the language switch [14] (but see Reference [6]), when univalent stimuli are employed [15] (but see Reference [16]), when participants switch language voluntarily rather than according to cues [17], or when testing early bilinguals who are highly proficient in both L1 and L2, even if the task requires them to switch between their strong L1 and a much weaker L3 [5,18]. These findings raise questions about the reliability of the asymmetrical switch cost as a marker for inhibition [19,20].

Another common finding from the language switching paradigm is the *reversed dominance effect*, sometimes called *global L1 slowing*. This refers to the overall slower naming latencies observed in L1 compared to L2 (on both stay and switch trials), a surprising occurrence given that naming in L1 should normally be faster than in L2 [21]. The reversed dominance effect is often interpreted as evidence for sustained inhibition of L1, which serves to facilitate L2 speech production in a mixed-language context [19,22,23]. It is interesting to note that, in studies where a switch cost asymmetry is absent, the reversed dominance effect is usually observed [5,12,14,17,18,24,25]. It seems to be a matter of whether the L1 slowing affects both stay and switch trials (i.e., reversed dominance) or only the switch trials (i.e., asymmetrical switch cost). From this point of view, switch cost asymmetry and reversed dominance serve as complementary evidence supporting the presence of inhibitory processes in language switching.

### 1.2. The Role of Domain-General Brain Mechanisms

In the past two decades, researchers began applying neuroimaging techniques to the study of bilingual language control. A growing amount of evidence now shows that language switching engages the brain network for executive control [26,27,28,29,30]. This gives rise to the idea that the neural mechanisms underlying language control may be similar to those underlying generic action control [31]. Based on such findings, Green and Abutalebi [32] developed a neurocognitive model of bilingual language control, which proposes a brain network of cortical and subcortical structures tightly related to executive function (see also Abutalebi and Green [10,33]).

In particular, the pre-supplementary motor area (pre-SMA) is often reported to be involved in language switching [29,34,35,36,37]. However, the exact pattern of pre-SMA activation varies across studies. For example, Garbin et al. [29] found that the pre-SMA was recruited only when bilinguals switched from their L2 into L1, not when switching in the other direction. In contrast, de Bruin et al. [36] report the opposite pattern in trilingual participants: pre-SMA activation occurred when participants switched into their L2 (or L3), but not when they switched into L1. Furthermore, Abutalebi et al. [35], who also tested trilingual participants, found that the pre-SMA was activated on all switch trials, regardless of which language the participants switched into. Such conflicting results leave open questions about exactly under what circumstances the pre-SMA is engaged and what its precise function is in language switching.

Outside of the linguistic domain, the pre-SMA is widely regarded as an important brain area in the inhibitory control network [38,39,40]. In recent years, it is increasingly recognised for its role in response selection and conflict resolution across domains, especially in demanding tasks [41]. The pre-SMA is sometimes considered as part of a complex with the dorsal anterior cingulate cortex (ACC). It is suggested that the pre-SMA may work together with dorsal ACC in carrying out conflict monitoring and error detection, in both language switching and non-linguistic tasks involving a high level of conflict [30,37]. In Green and Abutalebi’s neurocognitive model of bilingual language control [32], the role of performing conflict resolution belongs to the ACC/pre-SMA complex. However, a meta-analysis of fMRI studies on language switching [42] identified significant activation in midline pre-SMA but not in the ACC, suggesting that the pre-SMA portion of this structure may be more universally engaged in bilingual control.

In summary, existing neuroimaging studies provide strong evidence for the involvement of pre-SMA in bilingual language control. However, findings vary on exactly when this brain region is engaged. Furthermore, functional neuroimaging is unable to discern whether the pre-SMA plays a causal role in language switching or simply co-activates with the language control network. In the present study, we take the approach of creating a “virtual lesion”, by disrupting local brain activity in this region using non-invasive brain stimulation [43]. If this has an impact on language switching performance, then a causal relationship can be established between pre-SMA activity and language control. The role of the pre-SMA in language switching is generally regarded as conflict monitoring and resolution, without an exact specification of how such function is carried out (e.g., by biasing selection towards the target language, or by inhibiting the non-target language). Given that the pre-SMA acts as a key node in the brain network for domain-general inhibitory control, we hypothesise that it likely accomplishes conflict resolution via an inhibitory mechanism. In particular, we distinguish between two levels of inhibition in language switching (see below), and the pre-SMA may have a possible role in either or both of these. By examining whether each level of inhibition is affected by pre-SMA disruption, we might be able to pinpoint the mechanism via which this brain region carries out language control.

### 1.3. Two Levels of Language Inhibition

**Figure 1:**
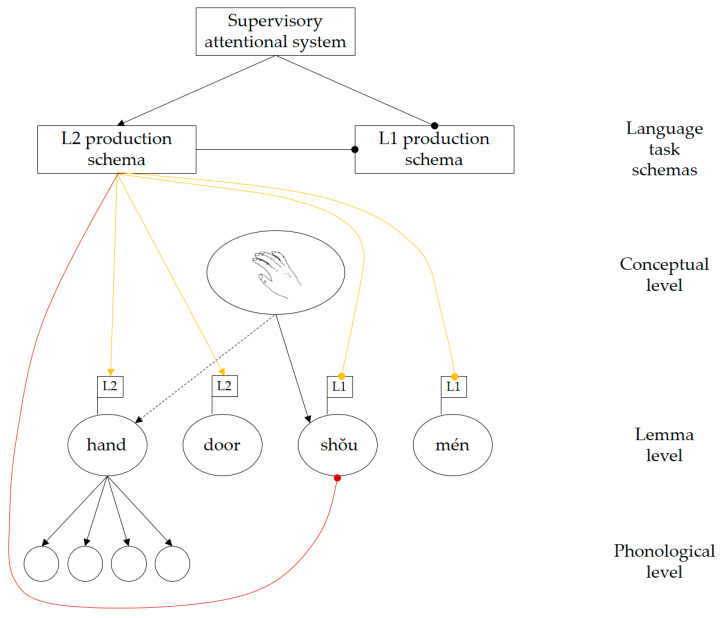
Whole-language and item-specific control in bilingual word production, based on Green [2] and De Groot and Christoffels [44]. Yellow lines indicate whole-language control; red lines indicate item-specific control. Arrow heads indicate excitation; circle heads indicate inhibition. In this example, the target language is L2 (English), and the non-target language is L1 (Mandarin). The intention to speak L2 is expressed by the supervisory attentional system, which activates the “L2 production schema” and suppresses the “L1 production schema”. The L2 production schema then applies whole-language control to regulate the activation levels of lemmas in the two languages, in order to bias selection towards L2 lemmas. The L1 lemma “shŏu” (translation-equivalent of the target name “hand”) receives strong activation from the concept and, thus, becomes a strong competitor despite being suppressed at the whole-language level. To ensure correct selection of the L2 lemma “hand” for output, the L2 production schema reactively inhibits “shŏu” to resolve the competition.

De Groot and Christoffels [44] propose that bilingual language control may operate at two different levels: *whole-language control* affects all lexical representations in a language simultaneously, whereas *item-specific control* targets specific lexical representations that are competing for selection (De Groot and Christoffels originally referred to these as “global” and “local” control; however, those terms have since been used with different definitions [45]. To avoid potential confusion, we adopt the less ambiguous terminology used by Van Assche et al. [46]). If both levels of control are present, proactive regulation on the whole-language level may be complemented by reactive inhibition operating at the item-specific level. According to Green [2], the intention to speak a particular language affects the activation levels of language task schemas (e.g., L1 production schema, L2 production schema). The active language task schema can then exert control on the whole-language level to bias selection towards lemmas in the target language. When a concept spreads activation to corresponding lemmas in both languages, the language task schema reactively inhibits any highly activated lemmas belonging to the non-target language, to ensure that speech output occurs in the desired language (see Figure 1). De Groot [47] argues that, in theory, item-specific control alone would be sufficient to prevent the output of any words belonging to the non-target language. Based on this view, whole-language control may be redundant.

Experimental studies so far examined these two possible levels of language control in a variety of paradigms. Philipp and Koch [48] asked participants to switch among three languages and investigated the “n-2 language repetition cost” which reflects inhibition of the recently abandoned language. They found that the repetition cost was similar regardless of whether the stimulus-response set or only the language was repeated, suggesting that such inhibition occurred on the whole-language level. Van Assche et al. [46] adopted a verbal fluency task, in which bilingual participants produced words beginning with certain graphemes/phonemes in each language, as specified by letter prompts. The task was conducted in a by-language blocked design. Difficulty of dominant language production when it occurred after the non-dominant language block was taken as evidence for inhibition of the dominant language. While item-specific inhibition (elicited by repeated prompts across languages) was found in both groups of bilinguals in that study, whole-language inhibition (elicited by non-repeated prompts) was only observed in Mandarin–English and not Dutch–English bilinguals, indicating that the latter might be an optional strategy. In blocked picture-naming tasks, the dominant language block suffered from slower responses following production of the non-dominant language, whether the picture stimuli were repeated across languages or not [49,50]. Dominant language production also recruited more cognitive resources when it occurred after the non-dominant language, whether the stimuli were repeated or not [45,51]. Such hindrance of dominant language production suggests the presence of sustained control on the whole-language level. In addition, Branzi et al. [51] observed activation of the dorsal-ACC/pre-SMA complex exclusively for naming of repeated stimuli in the non-dominant language, pointing towards a particular role of this brain region in item-specific control.

Taken together, these findings support the existence of whole-language and item-specific control in bilingual speech production, with potentially different underlying neural mechanisms. However, the presence of either or both levels of control in a particular situation seems to depend on the specific experimental paradigm used and the type of bilinguals tested. Given that, in the realm of language switching, a central piece of evidence for inhibitory control comes from the asymmetrical switch cost in the traditional cued-switching paradigm (i.e., trial-to-trial switching), it is important to examine the distinction between whole-language and item-specific inhibition within the context of this paradigm.

A previous attempt on this was made by Finkbeiner et al. [15], who investigated (what they called) the “language suppression hypothesis” and “lexical suppression hypothesis”. These hypotheses encompass a similar idea as whole-language vs. item-specific inhibition, but there are differences in the definitions. Specifically, the “lexical suppression hypothesis” suggests that naming a concept in one language suppresses all semantically related names in the other language (to a certain degree); this broad assumption may be one reason why such suppression was not observed in that study. In accordance with more recent studies (e.g., Branzi et al. [51]), we adopt a conservative definition of “item-specific inhibition”, which looks specifically at suppression of translation equivalents in the non-target language. Since direct translation-equivalents are likely to be the most potent competitors in lexical selection, this should elicit the strongest form of item-specific inhibition. In addition, Finkbeiner et al. [15] argued against both whole-language and item-specific inhibition, based on an absence of switch cost for the univalent stimuli in their study. However, this pattern was likely due to a confound of task switching which accompanied all univalent trials (such that the effect of language switching was masked by the task switch). More recently, Reynolds et al. [16] showed that the (typical) asymmetrical switch cost can indeed be obtained for univalent stimuli, when the confound of task switching is removed. Therefore, in the present study, we eliminate such confounds in the design. In the next section, we argue that the type of inhibition involved in trial-to-trial switching likely operates on the whole-language level, and we develop a comparable measure for item-specific inhibition so that the two levels of control can be examined in parallel. To maximise the opportunity for observing both whole-language and item-specific inhibition in this study, we target Mandarin–English bilinguals as they were previously shown to implement both levels of inhibition when another group of bilinguals did not [46].

### 1.4. The Present Study

The present study has two aims. The first aim is to examine whole-language and item-specific inhibition in the context of the traditional cued language-switching paradigm (Experiment 1). According to Branzi et al. [51], whole-language control is reflected in the after-effect of naming *any* item in the other language, whereas item-specific control is reflected in the after-effect of naming the *same* item in the other language. We now consider what type of inhibition is involved in *trial-to-trial switching* (i.e., comparing switch trials to stay trials). On a switch trial, the response language changes from the preceding trial, which means that any inhibition previously applied on this language needs to be overcome [2]. On a stay trial, the response language stays the same as the previous trial; therefore, no such processes are required. The difference between a switch trial and a stay trial rests on whether there is a language change, regardless of what individual lexical items are involved on these trials. Therefore, if there is any inhibition applied in this type of switching, it most likely operates on the whole-language level (for similar reasoning, see Finkbeiner et al. [15], Experiment 1). Note that the costs of carrying out other switch-related processes (e.g., cue encoding, task goal updating) may also contribute to the switch cost; however, if there is a cost associated with overcoming whole-language inhibition on switch trials, this should at least form one component in the switch cost. In particular, if the time it takes to overcome such inhibition differs significantly between the two languages (e.g., due to stronger inhibition applied on the dominant language, as Green [2] proposed), then we expect the switch cost to be asymmetrical.

To examine item-specific inhibition in a similar manner, we incorporate a novel element into the study design, which we shall refer to as *within-item switching*. This type of switching occurs when the same item is named in one language after being named in the other language. Under the hypothesis that item-specific inhibition is present in bilingual control, when an item is named in a particular language, its translation-equivalent should be strongly suppressed; therefore, when the latter subsequently becomes the target label on another trial, it will take extra time to overcome that prior inhibition. To index the cost of such item-specific inhibition, we look at the difference between two types of stimuli: univalent and bivalent^i^ (Appendix A). A *univalent stimulus* always requires a response in the same language every time it appears; a *bivalent stimulus* requires responses in different languages on different trials. Thus, univalent stimuli are analogous to “stay” trials (i.e., they stay in the same language as the last time this item was named, so there is no item-specific inhibition to overcome), while bivalent stimuli are analogous to “switch” trials (i.e., they change to a different language compared to the last time this item was named^ii^ (Appendix A), so any item-specific inhibition applied previously has to be overcome). As explained above, in trial-to-trial switching, the process of overcoming whole-language inhibition forms a part of the switch cost; by a similar logic, in within-item switching, the process of overcoming item-specific inhibition should be a component in the *valence cost* (i.e., difference between bivalent and univalent items). If the time it takes to overcome item-specific inhibition differs significantly for labels in the two languages (again, due to stronger inhibition applied on labels in the dominant language), then we expect the valence cost to be asymmetrical.

There is scant opportunity to examine the valence cost in the current language switching literature, as existing studies typically employ bivalent stimuli only. The few studies that used both univalent and bivalent stimuli in a language switching paradigm focussed on the question of whether the asymmetrical switch cost was uniquely found in bivalent stimuli [15,16], rather than directly looking into the difference between these two types of stimuli as an index for item-specific inhibition. In the present study, we ask bilinguals to perform a picture-naming task, in which univalent and bivalent stimuli are combined seamlessly. In this task, half of the stimuli are univalent, each consistently eliciting responses in the same language every time it appears; the other half are bivalent, each imposing varied language requirements throughout the experiment. Univalent and bivalent stimuli are mixed together and appear under exactly the same circumstances; therefore, the valence of each stimulus remains implicit in the eyes of the participants. This ensures that there is no confound between these two types of stimuli, thus allowing them to be compared directly (but see Section 4.3 for a possible improvement). By enabling such comparison between univalent and bivalent stimuli (i.e., a measurement of item-specific inhibition) alongside the comparison between stay and switch trials (i.e., a measurement of whole-language inhibition) within the same experimental task, the two levels of inhibition can be examined simultaneously and compared side by side.

The second aim of this study is to investigate the involvement of domain-general inhibitory control in language switching (Experiment 2). The motivation behind this is to verify the relevant theoretical accounts in neurocognitive models of bilingual language control [10,32,33] and to provide more concrete empirical basis for (or against) the view that language control relies on executive function. An increasing amount of neuroimaging evidence now suggests that language switching recruits the brain network responsible for domain-general inhibition, and chief among these brain areas is the pre-SMA. While neuroimaging findings can only reveal an association between pre-SMA activity and inhibitory control in language switching, the role of this brain region in language control will be further confirmed if a causal relationship can be established. In Experiment 2, we explore whether such a causal relationship exists, by externally disrupting the excitability of the pre-SMA region and then examining the consequence on bilinguals’ language switching performance. This disruption is achieved using a non-invasive brain stimulation technique called transcranial magnetic stimulation (TMS).

We examine the role of pre-SMA in language control with respect to the proposed distinction between whole-language and item-specific inhibition. As explained above, the present experimental design affords the ability to inspect both types of inhibition within the same task, thus providing an excellent opportunity to assess whether they share the same underlying neural mechanism. Experiment 1 aims to establish the presence of whole-language inhibition (as indexed by the switch cost) and item-specific inhibition (as indexed by the valence cost) in a picture-naming task, and then the same task is used in Experiment 2 to investigate whether either or both levels of inhibition are causally dependent on domain-general inhibitory control. Distinguishing between these two levels of control in language switching and examining the involvement of pre-SMA in each of them will provide more fine-grained information as to what exactly this brain region is responsible for in bilingual language control. This can help shed light on the specific role of the pre-SMA in language switching and inform future updates to neurocognitive models of bilingual speech production.

## 2. Experiment 1: Whole-Language and Item-Specific Inhibition in Language Switching

In this behavioural experiment, we aim to examine whole-language and item-specific inhibition side by side in a cued language switching paradigm. We ask bilingual participants to perform a picture-naming task, in which they switch between English and Mandarin according to a cue on each trial. In this task, half of the pictures have consistent language mappings throughout the experiment (i.e., univalent items), and the other half have changing language requirements (i.e., bivalent items). With such a design, the cost of whole-language inhibition can be examined when the language requirement changes from one trial to the next (i.e., switch vs. stay trials), and the cost of item-specific inhibition can be assessed when the same picture elicits a response in one language after having been named in the other (i.e., bivalent vs. univalent items). Based on the hypothesis that both whole-language and item-specific control are at play during language switching, we predict the following: (1) an asymmetrical switch cost, which indexes the time it takes to overcome whole-language inhibition on switch trials, and (2) an asymmetrical valence cost, which indexes the time it takes to overcome item-specific inhibition on bivalent trials.

### 2.1. Materials and Methods

#### 2.1.1. Participants

Sixteen healthy adult Mandarin–English bilinguals participated in this study for course credits or monetary compensation. One participant was excluded from all analyses due to voice key issues during the experiment (see Section 2.1.5 for more details), so the final sample included fifteen participants (seven males; mean age = 28.2 years). Bilinguals were required to be at least moderately proficient in both languages (a minimum self-rating of 4 out of 7, for each language). Participants were free from speech or language impairments, and all had normal or corrected-to-normal vision. Informed consent was obtained from all participants. The study was approved by the human ethics committee of Macquarie University (#5201200035).

Demographic information and language proficiency self-ratings were collected from all participants using a language history questionnaire (either completed at the end of the experiment or online in their own time). The Multilingual Naming Test (MINT [52]), a 68-item picture-naming test available in both English and Mandarin, was administered to each participant to obtain a more objective measurement of their language proficiency. The naming test was always given after the participant completed the experimental task, to avoid any possible influence on their performance. A summary of participant characteristics is presented in Table 1.

These participants acquired Mandarin at an early age in a home setting or at the beginning of primary school, and they started learning English halfway through primary school or from the beginning of high school. Three of the participants were slightly more dominant in English, while the rest were slightly more dominant in Mandarin^iii^ (Appendix A). The participants switched languages quite regularly in everyday life. It is worth noting that most Mandarin speakers also speak another variant of Chinese, so it was not practical to recruit “pure” Mandarin–English bilinguals. However, we only included participants for whom Mandarin and English were their two strongest languages.

#### 2.1.2. Materials

We selected eight black-and-white line drawings from the set of stimuli used in a previous language switching study [53]. The following items were included: *hand–shŏu, door–mén, tree–shù, horse–mă, pencil–qiānbĭ, bone–gŭtóu, king–guówáng, grapes–pútao* (the Mandarin names are presented here in hànyǔ pīnyīn–the romanisation system for spelling out Mandarin sounds). Each picture was to be named in English, Mandarin, or both in the experiment. Pictures were selected such that naming ambiguity (i.e., more than one possible name for a picture) was minimised in both languages, and no within-language or cross-language homophones existed among the 16 possible target names. All target names in English were either one- or two-syllable words that were 4–6 letters long, and all target names in Mandarin were one- or two-character words (in Mandarin, one character is one syllable). We ensured that there was minimal semantic relatedness between any two pictures, so that the sequence of pictures could be fully randomised without the risk of any semantic interference effects on naming latencies.

#### 2.1.3. Design and Procedure

**Figure 2:**
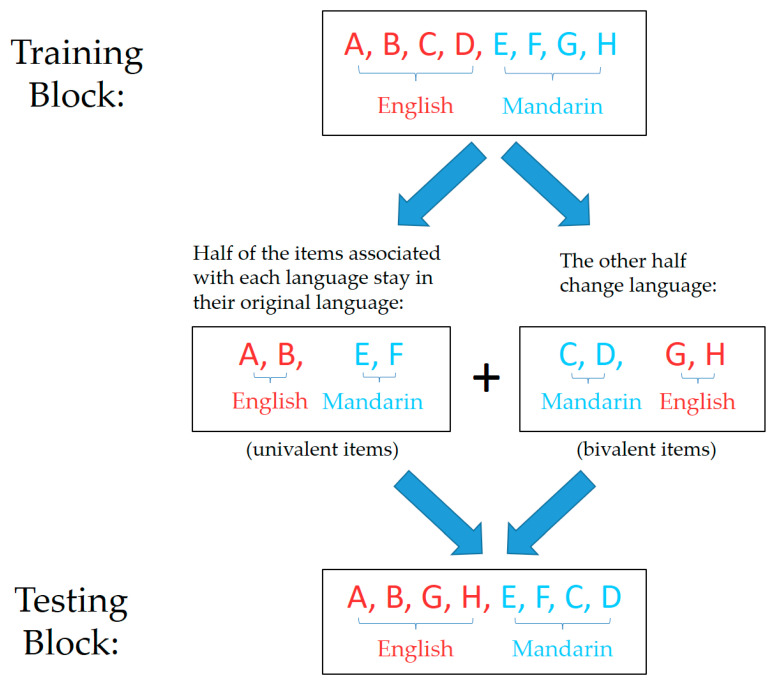
Illustration of the procedure used in Experiment 1 to achieve balanced assignments of language and valence to the picture stimuli. A total of eight pictures were used in this experiment. Here, each letter (e.g., “A”) represents one picture item. Items associated with English are shown in red; items associated with Mandarin are shown in blue. Univalent items maintained consistent language requirement in the two blocks, while bivalent items were trained and tested in opposite languages. Item–language pairings for the training block were randomly generated for each participant, such that four out of the eight pictures were associated with English and the other four with Mandarin (*top row*). Next, out of the four pictures associated with each language, two were randomly selected to be univalent and the other two were assigned to be bivalent. The language requirement for each bivalent item was changed (*middle row*). This produced the set of item–language associations to be used in the testing block (*bottom row*).

The picture-naming task was designed to allow a direct comparison between univalent and bivalent items (to examine item-specific inhibition) and between stay and switch trials (to examine whole-language inhibition). The task consisted of a training block and a testing block. Each univalent item maintained consistent language requirement throughout the two blocks, while each bivalent item was trained on one language and tested in the other. Item–language pairings were randomly generated for each participant when the experiment started, such that four out of the eight pictures were associated with English and the other four with Mandarin. Next, out of the four pictures associated with each language, two were randomly selected to be univalent and the other two were assigned to be bivalent. In the training block, the original item–language pairings were followed. In the testing block, those pictures that were assigned to be bivalent changed their language requirement (i.e., if it was originally trained in English, it now required naming in Mandarin, and vice versa), while the univalent pictures stayed in their original language (see Figure 2). The language requirement on each trial was specified using a language cue, which appeared simultaneously with the picture stimulus. The language cue was either “*what is this?*”, indicating the response was to be given in English, or the Chinese equivalent “*这是什么?*”, indicating a response in Mandarin was required. These language cues were designed to elicit responses in each language more naturally (compared to some commonly used cues, such as background colours or national flags), so as to minimise any cue-processing and related costs.

Participants were tested individually in a sound-attenuated room. Each session lasted approximately 35–45 min. The experiment was programmed in and controlled by the Presentation software (Version 18.3, Neurobehavioral Systems Inc., Berkeley, CA, USA). Stimuli were displayed on a Samsung SyncMaster SA950 (27 inch) monitor, connected to a Dell Optiplex 9010 PC (3.2 GHz Intel i5-3470 central processing unit, 8 GB random-access memory). Participants were seated comfortably in a chair 80 cm away from the monitor. Vocal responses were recorded through a microphone, and a voice key was set up in Presentation to detect response onset. The microphone amplifier volume was adjusted individually for each participant to optimise the functioning of the voice key. Before the picture-naming task commenced, participants were given verbal and onscreen instructions, which asked them to name the pictures as quickly and accurately as possible according to the language cue on each trial. Instructions were followed by a short practice block, which consisted of the same stimuli used in the experiment proper. Each stimulus appeared twice in the practice block. The purpose of the practice block was to allow participants to familiarise themselves with the task, as well as to make sure they had no trouble naming each picture. After a short break, participants initiated the training block themselves by pressing a key when they were ready. A short break was given after the training block, and then participants initiated the testing block, again by pressing a key themselves.

In the training block, each picture stimulus appeared 12 times. Pictures were presented in a random order for each participant, with the constraints that each picture appeared an equal number of times on stay trials and switch trials, and that no two consecutive trials had the same picture. In the testing block, trials were presented in the form of triads (i.e., groups of three), similar to the quartet structure used by Finkbeiner et al. [15]. In the triad structure, each (critical) trial was preceded by two filler trials. These two fillers always required responses in the same language, which served to ensure that each critical trial had a run-length of two (i.e., a switch trial would not directly follow another switch trial, which could result in a “stacked” effect). Thus, an example of a stay trial could be English→English→English, and a switch trial could be Mandarin→Mandarin→English. Each target picture stimulus appeared 12 times on critical trials (six stay trials and six switch trials), resulting in a total of 96 critical trials. As language and valence were already assigned earlier to all picture items in a random and balanced manner, this created critical trials that were fully balanced across language, valence, and transition type (stay vs. switch), eliminating possible bias due to factors other than the variables of interest. In addition, the same eight picture stimuli were used on the filler trials, so that each picture appeared 24 times as a filler. The fillers appeared no different to critical trials from the participants’ perspective, but they were not included in the data analysis. The triads were constructed in such a way that there was no repetition of pictures within each triad, and then all the triads were presented to the participant in a random sequence. The use of filler trials allowed dynamic sequences to be generated for each participant on the fly and further ensured participants would not be able to make predictions about the upcoming trial (as fillers were indistinguishable from critical trials). To avoid the naming of bivalent items on filler trials potentially overriding the training effect (and to maintain the bivalency throughout the testing block), filler trials used the original item–language pairings consistent with the training block. Thus, opposite languages were required on filler and critical trials for bivalent items^iv^ (Appendix A).

**Figure 3:**
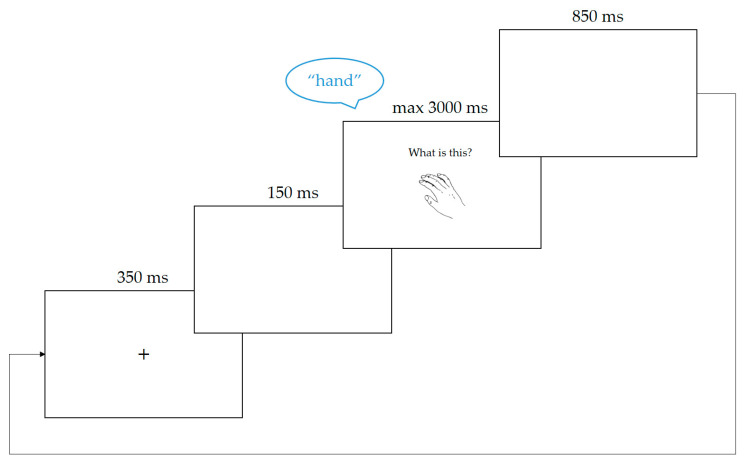
An example of a naming trial, showing the sequence of frames and the display of language cue (“what is this?”) and target stimulus (the picture of the hand). This example trial requires the response “hand” in English.

The trial structure is shown in Figure 3. Each trial started with a fixation cross which appeared at the centre of the screen for 350 ms. This was followed by a blank screen for 150 ms, before the language cue and picture stimulus appeared simultaneously on screen. The picture was displayed at the centre of the screen, while the language cue was located above it. Sound recording started as soon as the stimulus appeared. The trial was terminated upon the voice key being triggered by a response or 3000 ms after stimulus onset if no response was detected. The inter-trial interval lasted 850 ms, during which a blank screen was displayed, and then the next trial started. The vocal response on each trial was saved as an individual wave file for later verification.

#### 2.1.4. Offline Processing

The voice key in Presentation was triggered when the input speech volume from the microphone reached a certain threshold. This was intended to serve two purposes in the experiment: ending the current trial when a response was detected, and automatically reporting a reaction time (RT) value for each trial. While the speech detection was good enough for ending trials, the RT output (in milliseconds) did not reach the expected level of accuracy (i.e., the detected RTs did not consistently align with response onset across all trials). In order to obtain more accurate RT values, all of the wave files were processed offline using in-house software for speech onset detection. The detection output for each wave file was visualised as a graph and visually inspected to ensure accuracy. Any inaccurately detected RTs were identified, and those trials were subsequently excluded from the RT analysis.

Error coding was performed manually for all trials by checking the sound recording against the target response. The definition of “error” used here was a broad one, which included incorrect responses, as well as all verbal disfluencies (e.g., partial responses, stuttering, and utterance repairs). If the participant started giving the correct response but hesitated before having sounded out the complete word, or if they started to make a mistake but quickly corrected themselves, these were all counted as error trials. In other words, only straightforward correct responses were scored as correct. The reasoning is that those disfluencies represent cases of high conflict (which we are interested in for the same reason that we are interested in error trials), and determining which of these trials should be classified as correct and which as error often must involve subjective interpretation of the response given.

One participant was excluded from all data analyses due to heavy breathing triggering the voice key on a large number of trials. Even though this did not affect the RT values (as speech onsets were correctly detected by the post-processing procedure described above), the early triggering of voice key meant that the trial ended (and stimulus disappeared) before an actual response was produced. This could affect the RT for the current trial in unknown ways. Moreover, the early ending of trials resulted in shortened inter-trial interval (which started as soon as each trial ended), and it appeared that there was insufficient time following these trials for the participant to get ready for the upcoming trial.

#### 2.1.5. Data Analysis

All error trials were excluded from the RT analysis. We also excluded the trials identified earlier with inaccurately detected RTs, and trials with RTs outside 2.5 SD of each participant’s mean. Statistical analyses were performed in R (Version 3.4.4, R Foundation for Statistical Computing, Vienna, Austria) [54] using the “lme4” package [55]. The RT and error data were submitted to 2 × 2 × 2 linear mixed-effects models with the following factors: “valence” (univalent vs. bivalent items), “transition type” (stay vs. switch trials), “language” (L1 vs. L2), and the interactions between them were included as fixed effects; “participant” and “item” were included as random effects. For any follow-up tests conducted to unpack interactions, the *p*-values were adjusted using Bonferroni correction. Effects were categorised as significant at *p* < 0.05 and marginally significant at *p* < 0.1.

The analysis of RT data was conducted using both the raw values and log-transformed values. The latter was an attempt to satisfy the “normality of residuals” assumption of the linear model; however, the resulting model did not seem to meet this requirement (Shapiro–Wilk test: *p* < 0.0001; Kolmogorov–Smirnov test: *p* < 0.0001). It is argued that log transformation does not always achieve such purpose, in which case it may be more appropriate to apply statistical methods that do not come with these assumptions [56]. Therefore, we also conducted permutation tests to estimate the *p*-values for the model terms. This is a popular non-parametric method which does not assume any particular underlying data distribution [57]. The permutation tests were conducted on RT data using the “permanova.lmer” function in the R package “predictmeans” [58]. All versions of analysis (raw RT, log-transformed RT, and permutation tests) agreed in terms of which effects were significant and which were not, but the exact statistical values differed. We report the raw RT version in the in-text description below and include all versions of results in Appendix B.

### 2.2. Results

Following the trial exclusion procedure described above, approximately 10.7% of trials were excluded from the RT analysis. Mean reaction time and error rate in each condition are shown in Table 2. Statistical analyses were performed on single-trial RT and error data.

The RT analysis revealed significant main effects of valence, transition type, and response language. Bivalent items were named more slowly than univalent items (mean difference 165 ms; *χ*^2^ (1) = 215.7919, *p* < 0.0001). Responses were slower on switch trials compared to stay trials (19 ms; *χ*^2^ (1) = 6.6664, *p* = 0.0098). Naming in L1 took longer than in L2 (60 ms; *χ*^2^ (1) = 27.7909, *p* < 0.0001). Importantly, there was a significant interaction between valence and language, such that the RT difference between bivalent and univalent items (i.e., valence cost) was larger in L1 (218 ms) than in L2 (116 ms): *χ*^2^ (1) = 9.1807, *p* = 0.0024. Follow-up tests revealed that the valence cost was significant within each language (L1: *t* = 12.203, *p* < 0.0001; L2: *t* = 8.477, *p* < 0.0001). The *p*-values in follow-up tests were adjusted for multiple comparisons using Bonferroni correction. No other interactions were significant in the RT analysis.

The error analysis showed significant main effects of valence and response language. Participants made more errors on bivalent items compared to univalent items (mean difference 13.6%; *χ*^2^ (1) = 56.2991, *p* < 0.0001), and they made more errors when responding in L1 compared to L2 (3.3%; *χ*^2^ (1) = 5.0815, *p* = 0.0242). The main effect of transition type was not significant in the error analysis, nor were any of the interactions between factors.

### 2.3. Discussion

In this experiment, we aimed to examine whole-language inhibition (via trial-to-trial switching) and item-specific inhibition (via within-item switching) side by side in a cued language switching task. The main effects of transition type and valence were significant, demonstrating that switch trials were more difficult than stay trials, and naming bivalent items was more difficult than naming univalent items. As explained earlier (see Section 1.4), the presence of switch cost and valence cost alone does not necessarily mean there is inhibition involved. The switch cost may capture other processes related to switching (e.g., cue encoding, task goal updating), and the valence cost may reflect other differences between univalent and bivalent items (e.g., the target labels for univalent items might benefit more from repetition priming, since they were repeated more frequently than the target labels for bivalent items; see Section 4.3 for a more detailed discussion on this). A key marker for inhibition would be identified if these costs were asymmetrical between the two languages.

**Figure 4:**
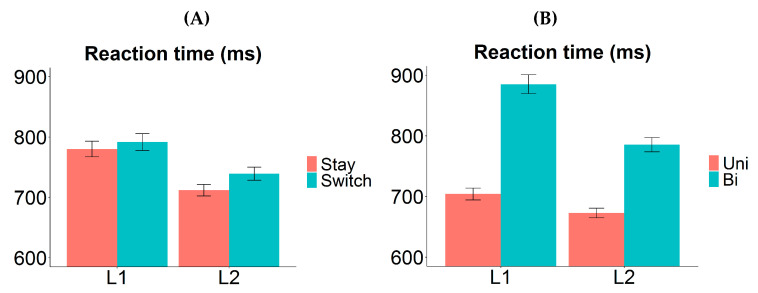
Switch cost and valence cost in Experiment 1. (**A**) Reaction time as a function of “response language” (L1 vs. L2) and “transition type” (stay vs. switch trials). The switch cost was symmetrical between the two languages. (**B**) Reaction time as a function of “response language” (L1 vs. L2) and “valence” (univalent vs. bivalent items). The valence cost was significantly larger in L1 compared to L2. Error bars indicate one standard error above and below the means. Uni = univalent items; Bi = bivalent items.

Here, we observed an asymmetrical valence cost, where the RT difference between bivalent and univalent items was found to be larger in L1 (Figure 4B). The valence cost was nonetheless significant in L2. The most straightforward explanation for the asymmetry is that stronger item-specific inhibition was applied on the competing L1 labels when bivalent items were named in L2 than vice versa; as a result, it took longer to overcome the prior item-specific inhibition when bivalent items were to be named in L1 again. Other possible components of the valence cost are unlikely to generate such an asymmetry. For example, differential amount of repetition priming may cause univalent items to be named faster and more accurately than bivalent items, but such repetition should benefit L1 and L2 equally (i.e., the valence effect should not interact with language); alternatively, it may benefit the L2 more (as that is the less practiced language), in which case the valence cost should be larger for L2. These possibilities are inconsistent with our observation of a larger valence cost in L1.

Interestingly, we observed symmetrical switch cost between the two languages (Figure 4A). This suggests that either whole-language inhibition did not occur in trial-to-trial switching, or the strength of the whole-language inhibition was similar between L1 and L2. Given that our participants were relatively proficient in their L2 (compared to most studies where switch cost asymmetry was found), the latter is likely to be true. Moreover, we observed a reversed dominance effect, i.e., L1 naming was overall significantly slower than L2 naming (on both stay and switch trials). Such a pattern is usually found in cases where the switch cost asymmetry is absent [5,14,17,18,24], and it is often interpreted as evidence for sustained inhibition of L1 in a mixed-language production context [19,22,23]. Note though that the reversed dominance effect here may be driven entirely by the bivalent items (see Figure 4B); thus, it should be interpreted with caution. We defer further discussions about this to Section 4.1.

## 3. Experiment 2: Domain-General Inhibitory Control in Language Switching

In Experiment 1, we examined whole-language and item-specific inhibition in a picture-naming task with cued language switching. We set out to investigate whether both types of inhibition were involved in this task and, if so, to find a behavioural index for each. We observed an asymmetrical valence cost as predicted, suggesting that item-specific inhibition was present, and that the amount of inhibition applied on L1 labels was stronger than on L2 labels. On the other hand, symmetrical switch cost was observed between the two languages. While this could be explained by a complete lack of whole-language inhibition, it is more likely that the inhibition on both languages were simply of similar strength (see discussions in Section 2.3). In this case, whole-language inhibition would still be a component in the switch cost.

In this experiment, we investigate the neural mechanisms underlying whole-language and item-specific inhibition. In particular, we are interested in whether one or both of them engages domain-general inhibitory control. De Groot and Christoffels [44] propose that these two types of control operate at different times and serve distinct purposes in lexical selection; therefore, it is likely that they operate via different neural mechanisms. We examine a particular brain area known for its role in domain-general inhibitory control: the pre-SMA. This brain region is frequently found to activate during language switching, which suggests its possible involvement in performing inhibition in language control. However, it remains unclear whether the pre-SMA plays a causal role in language inhibition and, if so, what its precise function is. Using the same picture-naming task as Experiment 1, we investigate these questions by perturbing the pre-SMA via a repetitive TMS protocol and observing the effect on whole-language and item-specific inhibition. As explained in Section 1.4, whole-language inhibition forms a component of the switch cost, while item-specific inhibition forms a component of the valence cost. If the pre-SMA plays a causal role in either type of inhibition, then TMS should modulate the corresponding type of cost (or the asymmetry of it). This would allow us to pinpoint which level of language control relies on the pre-SMA and infer more precisely the role of this brain region in language switching. Based on previous findings from a different paradigm [51], we predict that the pre-SMA has a more prominent role in item-specific control.

### 3.1. Materials and Methods

#### 3.1.1. Participants

Sixteen healthy adult Mandarin–English bilinguals (five males; mean age = 24.6 years) participated in this study for course credits or monetary compensation. All participants were right-handed; they were free from any neurological disorders and met the safety requirements for undergoing MRI and TMS. Participants were not taking any psychiatric medication, and all had normal or corrected-to-normal vision. Each participant gave informed consent before taking part in the experiment. The study was approved by the human ethics committee of Macquarie University (#5201400585).

Individual high-resolution T1-weighted brain MRI images were obtained for each participant for the purpose of localising the target area for TMS. Each participant was then tested in two separate TMS sessions, which were scheduled at least one week apart. The TMS sessions were all scheduled in the afternoon, and the two sessions for the same participant always took place around the same time of day (with at most one hour of difference between the starting times) to minimise possible influence of circadian rhythm on the efficacy of TMS [59]. Testing order was fully counterbalanced in regard to TMS order (pre-SMA stimulation in first session, or control site in first session).

Demographic information and language proficiency self-ratings were collected from all included participants via a language history questionnaire. The MINT test [52] was also administered to each participant to obtain a more objective assessment of their proficiency in each language; this test was always done at the end of the second TMS session, to avoid having any possible influence on their performance during the experimental tasks. A summary of participant characteristics is presented in Table 3.

#### 3.1.2. Target Localisation

The pre-SMA is a small cortical region located on the medial frontal cortex (very close to the midline between the two hemispheres of the brain). The fMRI studies that identified activation of this area in language switching simply referred to it as “pre-SMA” (without stating whether it is the left or right side), and a meta-analysis summarised this as “midline pre-SMA” [42]. However, a precise target location is required for TMS, as stimulating on the midline (i.e., on top of the medial longitudinal fissure) would likely result in ineffective stimulation of either the left or right pre-SMA. The right pre-SMA was chosen in this study because it is more commonly accepted as part of the inhibitory control network [60].

**Figure 5:**
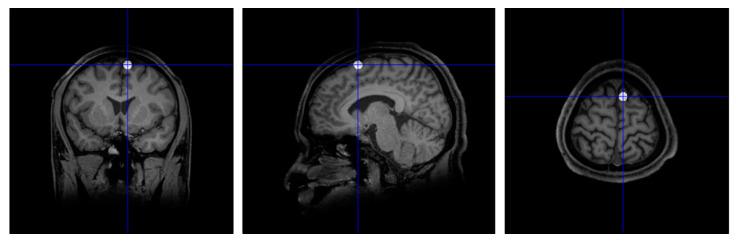
The position of right pre-supplementary motor area (pre-SMA) marked on the individual MRI scan.

A high-resolution T1-weighted structural brain MRI scan (slice thickness: 1 × 1 × 1 mm) was obtained for each participant (Macquarie Medical Imaging, Macquarie University Hospital, Sydney). The images were firstly reoriented as necessary such that the head was upright and the anterior commissure (AC) and posterior commissure (PC) were on the same horizontal line. The pre-SMA was then located anatomically, using a procedure similar to that described by Tremblay and Gracco [61]. We adapted this procedure to locate the right rather than the left pre-SMA. A vertical line was drawn 10 mm anterior to the AC, forming a coronal plane which intersects the cerebral cortex at the top. The right pre-SMA was identified as a point along the intersection on the medial most portion of the right superior frontal gyrus (SFG). The coordinates of this point were noted, and a white spherical blob was drawn onto the MRI at this position using an in-house Matlab script (Figure 5).

Localisation of TMS target on the participant was guided by a frameless stereotaxic system (Visor2, ANT Neuro, Enschede, the Netherlands; http://www.ant-neuro.com). The MRI images for each individual participant were loaded into the navigation system and a three-dimensional (3D) model of the head and brain was reconstructed from these images. The target location was then marked in the system at the location of the white blob drawn earlier. During each TMS session, an MRI co-registration procedure was performed to link the 3D model to the participant’s head in real space. The participant wore a headband with reflective spherical markers, which were tracked by the navigation system. The navigation system then guided the placement of the coil over the predefined target location.

The vertex, which served as the control site, was defined as the halfway point between the nasion and inion [60]. This location was determined with tape measurement, and the desired coil position was marked for later use. For both stimulation sites, the coil was held with the handle pointing in the posterior direction. The same MRI co-registration procedure and tape measurement were carried out during both the experimental session and the control session to make the two sessions appear identical from the participant’s perspective, and participants were told that two areas of interest were being investigated. During the debriefing at the end of the entire study, participants reported similar sensations from TMS during both sessions, and some expressed surprise upon learning that one of these sessions was the control condition. When asked to guess which session was experimental and which was control, they were unable to tell (more than half gave the incorrect answer).

#### 3.1.3. TMS Procedure

Magnetic stimulation was delivered using a Magstim Rapid2 stimulator (Magstim Co., Whitland, UK), with a handheld 70-mm figure-of-eight coil. Resting motor threshold (RMT) was determined individually for each participant. The RMT was defined as the minimum intensity applied on the right primary motor cortex (M1) to elicit three visible twitches on the contralateral first dorsal interosseous (FDI) muscle out of five consecutive stimuli. Participants were instructed to keep their hand muscles relaxed while the RMT was determined.

Continuous theta-burst stimulation (cTBS) [62] was used to achieve transient suppression of the right pre-SMA. This is a repetitive TMS protocol capable of inducing a reduction of cortical excitability thought to be mediated by long-term-depression-like mechanisms [63]. The suppressive effect of cTBS on pre-SMA excitability was previously demonstrated [64]. In the cTBS protocol, each burst consisted of three pulses delivered at 50 Hz, and the bursts were repeated at 5 Hz. As such, a total of 600 pulses were delivered over a period of 40 s. In accordance with previous studies [65], stimulation intensity for each individual was calculated as 80% of their RMT. The participants in this study had rather high RMT in general (this may be race-related, see Yi et al. [66]). Due to capacity limit on the stimulator, the maximum output intensity achievable in the cTBS protocol was 51% (we discuss this further in Section 4.3).

#### 3.1.4. Behavioural Task

The behavioural task was the same picture-naming task used in Experiment 1, with identical materials and procedure. After the RMT was determined and MRI co-registration was performed, the participant was given verbal and onscreen instructions for the task and completed the first part of picture naming (i.e., the training block). The co-registration accuracy was checked (by validating the nasion position) immediately before TMS to ensure the navigation markers worn on the participant’s head did not move relative to the head (in one case where the validation failed, the co-registration procedure was carried out again before TMS). Then, cTBS was delivered for 40 s and the participant was instructed to rest for 5 min without talking. This waiting time was based on observations on the after-effects of cTBS over M1, where the modulation of motor evoked potentials (MEP) was found to be most reliable at 5 min post-stimulation [67]. After the 5-min waiting time, the participant was instructed to proceed to the second part of picture naming (i.e., the testing block).

To make the results from the two TMS sessions more comparable and to ensure there were no contradicting training effects during the two sessions, the same item–language pairings and item–valence assignment were maintained for each individual. In the first TMS session, the pairings were randomly generated just as in Experiment 1; in the second session, the previously generated pairings were used instead of new pairings being created.

#### 3.1.5. Data Analysis

The procedures for offline RT detection, error coding, and trial exclusions were identical to Experiment 1. Each participant underwent two test sessions in this experiment (TMS stimulation on pre-SMA and vertex); thus, a new factor was introduced into the analysis. RT and error data were submitted to 2 × 2 × 2 × 2 linear mixed-effects models: “TMS location” (pre-SMA vs. control site), “valence” (univalent vs. bivalent items), “transition type” (stay vs. switch trials), “language” (L1 vs. L2), and the interactions between these were included as fixed effects; “participant” and “item” were included as random effects. Effects were categorised as significant at *p* < 0.05 and marginally significant at *p* < 0.1.

As with Experiment 1, the analysis of RT data was conducted using both the raw values and log-transformed values. Since the “normality of residuals” assumption of the linear model was not satisfied even after log transformation, we again conducted permutation tests as an additional check. The results from the analyses of raw RT and log-transformed RT agreed on all effects, except a marginal interaction which was only present in the raw RT version. In the permutation tests, this interaction was found to be marginally significant (i.e., agreeing with the raw RT results). In the section below, we report the statistical values from the raw RT analysis, and we include all versions of results in Appendix C.

### 3.2. Results

Following the trial exclusion procedure described in Experiment 1, approximately 16.3% of trials were excluded from the RT analysis. Mean reaction time and error rate in each condition are shown in Table 4. Statistical analyses were performed on single-trial RT and error data.

The RT analysis revealed significant main effects of TMS location, valence, transition type, and response language. Perturbation of the pre-SMA resulted in longer naming latencies compared to control site stimulation (mean difference 21 ms; *χ*^2^ (1) = 10.2989, *p* = 0.0013). Bivalent items were named more slowly than univalent items (149 ms; *χ*^2^ (1) = 516.1176, *p* < 0.0001). Responses were slower on switch trials compared to stay trials (13 ms; *χ*^2^ (1) = 5.8391, *p* = 0.0157). Naming in L1 took longer than in L2 (12 ms; *χ*^2^ (1) = 9.4246, *p* = 0.0021). As in Experiment 1, there was a significant interaction between valence and language, such that the RT difference between bivalent and univalent items (i.e., valence cost) was larger in L1 (156 ms) compared to L2 (143 ms): *χ*^2^ (1) = 5.1166, *p* = 0.0237. Follow-up tests revealed that the valence cost was significant within each language (L1: *t* = 17.558, *p* < 0.0001; L2: *t* = 14.878, *p* < 0.0001). There was also a marginally significant interaction between valence and TMS location, with the valence cost being larger when pre-SMA was perturbed (160 ms) compared to control site (137 ms): *χ*^2^ (1) = 3.5719, *p* = 0.0588. Follow-up tests revealed that the valence cost was significant in each condition (pre-SMA: *t* = 17.740, *p* < 0.0001; control site: *t* = 14.952, *p* < 0.0001). The *p*-values in follow-up tests were adjusted for multiple comparisons using Bonferroni correction. No other interactions were significant in the RT analysis.

The error analysis showed significant main effects of valence, transition type, and response language. Participants made more errors on bivalent items compared to univalent items (mean difference 23.7%; *χ*^2^ (1) = 233.9853, *p* < 0.0001), more errors on switch trials than stay trials (2.3%; *χ*^2^ (1) = 3.9657, *p* = 0.0464), and more errors when responding in L1 compared to L2 (4.2%; *χ*^2^ (1) = 10.5175, *p* = 0.0012). There was also a three-way interaction betweem TMS location, transition type, and language: *χ*^2^ (1) = 7.0611, *p* = 0.0079. Follow-up tests showed that there was no switch cost asymmetry (i.e., two-way interaction between transition type and language) either when the pre-SMA was perturbed (*z* = 1.430, *p* = 0.1528) or when the control site was perturbed (*z* = 0.398, *p* = 0.6909). No other main effects or interactions were significant in the error analysis.

### 3.3. Discussion

The purpose of this experiment was to investigate the involvement of domain-general inhibitory control in language switching. In particular, we examined whether the pre-SMA had an essential role in whole-language and/or item-specific inhibition. Using the same behavioural task as Experiment 1, bilingual participants named pictures and switched between English and Mandarin according to the language cue on each trial. Participants’ performance from the two TMS sessions (pre-SMA and vertex perturbation) were compared to see if the disruption of pre-SMA activity had any impact on language switching. The cTBS protocol was intended to induce a reduction of cortical excitability at the stimulation site, resulting in a suppressive effect on that brain region. The use of a control site (vertex) as baseline allowed a direct examination of the consequence of target site (pre-SMA) stimulation, without the risk of the observed effect being an artefact (e.g., merely a generic effect of applying TMS).

**Figure 6:**
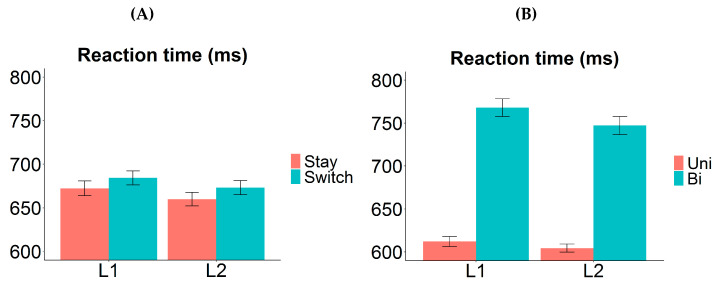
Switch cost and valence cost in Experiment 2. (**A**) Reaction time as a function of “response language” (L1 vs. L2) and “transition type” (stay vs. switch trials). The switch cost was symmetrical between the two languages. (**B**) Reaction time as a function of “response language” (L1 vs. L2) and “valence” (univalent vs. bivalent items). The valence cost was asymmetrical (larger in L1). Error bars indicate one standard error above and below the means. Uni = univalent items; Bi = bivalent items.

All the findings from Experiment 1 were replicated here, including the behavioural indices for whole-language and item-specific inhibition. The main effects of both valence and transition type were significant, signifying the presence of a switch cost and a valence cost. The valence cost was asymmetrical between the two languages (larger in L1), suggesting that there was stronger item-specific inhibition applied on L1 labels compared to L2 labels (Figure 6B). While the switch cost was symmetrical, we again observed a reversed dominance effect (Figure 6A), which is an indication of sustained inhibition of L1 (see Section 2.3 for detailed discussions on these interpretations).

The disruption of pre-SMA activity using TMS had an overall impact on participants’ performance. Naming on all trials was slowed by the perturbation of pre-SMA (compared to control site). This observation aligns with the reported involvement of this brain region in word selection and speech execution in general [68,69,70]. We attempted to pinpoint the role of the pre-SMA in bilingual control by assessing how its disruption affected the switch cost and the valence cost. According to the rationale laid out earlier, if the pre-SMA plays a causal role in item-specific inhibition (which is indexed by a component in the valence cost), then TMS should modulate the valence cost or its asymmetry between the two languages. Similarly, if the pre-SMA plays a causal role in whole-language inhibition (which is indexed by a component in the switch cost), then TMS should modulate the switch cost or its (lack of) asymmetry. We discuss the relevant findings below.

**Figure 7:**
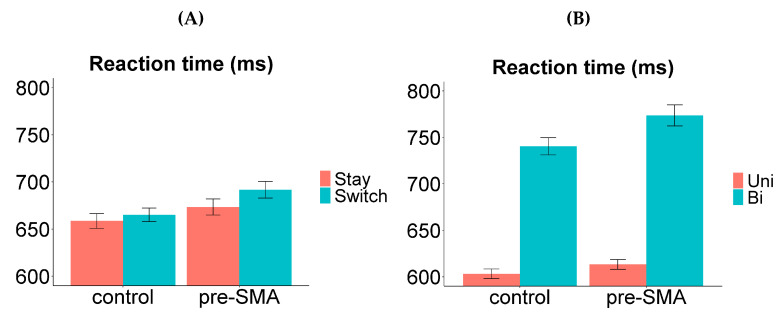
The effect of TMS stimulation on the switch cost and valence cost in Experiment 2. (**A**) Reaction time as a function of “TMS location” (control site vs. pre-SMA) and “transition type” (stay vs. switch trials). TMS did not significantly modulate the switch cost. (**B**) Reaction time as a function of “TMS location” (control site vs. pre-SMA) and “valence” (univalent vs. bivalent items). The valence cost was larger when TMS was applied on the pre-SMA (marginally significant interaction). Error bars indicate one standard error above and below the means. Uni = univalent items; Bi = bivalent items.

There was a marginally significant interaction between TMS location and valence, with the valence cost being larger when the pre-SMA was perturbed (Figure 7B). This indicates a possible role of the pre-SMA in item-specific inhibition, which is consistent with Branzi et al. [51], who suggest that the dorsal-ACC/pre-SMA complex is specifically recruited to handle increased monitoring demands in item-specific control. If this interaction is real, what did the disruption of pre-SMA actually affect? The fact that the valence cost increased (rather than reduced) with pre-SMA disruption demonstrates that this intervention did not simply make the item-specific inhibition weaker (if it did, the weaker inhibition should take less time to overcome, resulting in a smaller valence cost). Instead, the present findings suggest that pre-SMA perturbation may have affected another process represented by the valence cost (alongside overcoming item-specific inhibition). It was previously shown that disrupting the activity of pre-SMA could slow down the inhibition process such that it takes more time to complete successfully [71]. Therefore, after TMS was delivered over this brain region, it might have taken longer to achieve the appropriate level of item-specific inhibition on each bivalent trial to prevent erroneous output, resulting in an increased valence cost. In addition, the asymmetry of the valence cost was not affected by pre-SMA disruption, showing that competing L1 labels (on L2 bivalent trials) were still suppressed more strongly than vice versa, and the strong suppression took longer to overcome subsequently. It is important to note that the interaction was only marginally significant; therefore, the interpretation above should be taken with caution.

There was no interaction between TMS location and transition type in the naming latencies. In other words, the switch cost did not show a significant change when TMS was applied on the pre-SMA compared to the control site (Figure 7A). A three-way interaction was found between TMS location, transition type, and language in the error analysis, showing a trend of eliminating switch cost asymmetry when the pre-SMA was perturbed. However, follow-up tests revealed no significant switch cost asymmetry either under pre-SMA disruption or control site disruption; hence, the interaction was likely driven by the change of direction in the switch cost (i.e., it was slightly larger in L1 under control site perturbation, and slightly larger in L2 under pre-SMA perturbation). This pattern was only observed in the error data.

In summary, we observed an essential role of the pre-SMA in general speech production, but we did not find strong evidence for a causal role of this brain region in either whole-language or item-specific inhibition. While there is some indication of its involvement in these two levels of control (i.e., marginally significant modulation of RT valence cost, and modulation of switch cost asymmetry in error data), this is inconclusive evidence and should, therefore, be interpreted with caution.

## 4. General Discussion

The present study aimed to answer two questions about language control in bilingual speech production. The first question was in regard to whether both whole-language and item-specific inhibition were involved in language switching. We examined these in parallel in a modified language switching paradigm (Experiment 1). Item-specific inhibition (indexed by the valence cost) was more pronounced in the dominant language, indicating stronger inhibition of the individual labels in L1 than L2; whole-language inhibition (indexed by the switch cost) was symmetrical between the two languages. The valence cost was much larger in magnitude compared to the switch cost, suggesting that the strength of item-specific inhibition may be greater than whole-language inhibition (this difference could also be caused by other components in the switch cost and valence cost, see Section 4.3 for further discussion).

The second question concerned whether brain mechanisms for domain-general inhibitory control played an essential role in whole-language and/or item-specific inhibition. [31] We employed a repetitive TMS protocol to disrupt the functioning of the pre-SMA, a prominent region in the inhibitory control brain network (Experiment 2). Such disruption led to an overall slowing of naming latencies, suggesting a general role of the pre-SMA in speech execution. However, we did not find reliable evidence for its causal involvement in either whole-language or item-specific inhibition in the coordination of two languages. Given the lack of reliable modulation of either the switch cost or the valence cost by pre-SMA perturbation, we focus on patterns in the behavioural results in the discussions below.

### 4.1. Item-Specific Inhibition and the Reversed Dominance Effect

One interesting phenomenon in the current language switching literature is that the involvement of inhibition is underpinned by two distinct pieces of evidence, which occur in a somewhat complementary manner (see Section 1.1). Specifically, some studies report an asymmetrical switch cost [3,4,6,7,8,11,13], while others report a reversed dominance effect [5,12,14,17,24,25]. The observation of either of these patterns is generally taken as evidence for the presence of inhibitory processes in language switching. However, at this stage, there is no clear theory about why (or when) one or the other pattern would emerge in a particular situation. In the present study, the inclusion of two different types of stimuli (univalent and bivalent) provides a unique opportunity to look into possible differences in the mechanisms underlying the asymmetrical switch cost and reversed dominance effect, especially in regard to the level of control these mechanisms operate at. This might help provide a preliminary answer as to why some studies observe one pattern while other studies observe the other pattern.

In both of the experiments reported here, we found a significant interaction between stimulus valence (univalent vs. bivalent) and language (L1 vs. L2). In the analyses and discussions so far, we interpreted this interaction as a reflection of differential amount of item-specific inhibition applied on L1 and L2 labels. In other words, the “valence cost” was different between the two languages. Now, we present an alternative angle to look at this interaction—the reversed dominance effect (i.e., global slowing of L1) was different between univalent and bivalent items. Post hoc analyses conducted separately on bivalent and univalent stimuli (with Bonferroni correction) show that the L1 slowing affected bivalent items (*t* = 3.709, *p* = 0.0004) but not univalent items (*t* = 0.854, *p* = 0.7867). Given the usual interpretation of the reversed dominance effect as evidence for sustained L1 inhibition in a mixed-language production context to facilitate L2 output [19,22,23], the fact that such inhibition of L1 only impacted the bivalent items makes an interesting suggestion, i.e., the global slowing^v^ (Appendix A). observed was due to inhibition of individual lexical items rather than the entire lexicon. If the sustained inhibition affected L1 as a whole, then bivalent and univalent items should be slowed down by a similar degree. The present findings suggest otherwise: items that were previously named in L2 suffered extra slowing when being subsequently named in L1, whereas items that were not previously named in L2 (within the context of this experiment) did not suffer from slowing when named in L1.

Following this logic, we predict that it should not be possible to obtain a reversed dominance effect using univalent stimuli (i.e., if stimuli are never repeated, or if each stimulus maintains a fixed language association every time it appears). Consistent with this prediction, all language switching studies that observed a reversed dominance effect so far utilised bivalent stimuli. Notably, Kleinman and Gollan [53] compared cued switching (bivalent with forced language selection), bottom-up switching (univalent with free language selection), and voluntary switching (bivalent with free language selection), using the same set of stimuli. The reversed dominance effect only disappeared in the bottom-up switching block and only when this block occurred first (i.e., each stimulus was not yet named in the other language; hence, there was no item-specific inhibition to overcome). It remains to be seen whether any future studies employing univalent stimuli would be able to refute the prediction above.

If the reversed dominance effect results from item-specific inhibition, then what about asymmetrical switch cost? Theoretically, the latter should reflect whole-language inhibition, as the difference between a switch trial and a stay trial rests on whether there is a language change, regardless of what individual lexical items are named. It follows that any switch cost asymmetry should not be affected by stimulus valence (since whole-language inhibition should be applicable to both univalent and bivalent stimuli). Indeed, we observed no evidence of stimulus valence modulating switch cost asymmetry in the two experiments reported here^vi^ (Appendix A). This differs from the findings by Finkbeiner et al. [15], where asymmetrical switch cost was observed for bivalent stimuli, but no switch cost was observed for univalent stimuli. However, as Abutalebi and Green [33] noted, all the univalent trials in that study were accompanied by a task switch, which might have masked the language-switch effect. When the confound of task switching is removed, it seems that asymmetrical switch cost can occur in both univalent and bivalent stimuli [16].

In sum, the commonly reported behavioural markers of inhibition in language switching—asymmetrical switch cost and reversed dominance effect—seem to arise from language control operating at different levels. While the former reflects the effect of whole-language inhibition, the latter reflects the effect of item-specific inhibition. In regard to what factors determine whether the switch cost asymmetry or reversed dominance would be observed in a given situation, we speculate that one major factor is the type of participants tested. Late bilinguals (who are usually more unbalanced) tend to keep their two languages separate; thus, they need to apply control on the whole-language level to regulate the activation of each language. On the other hand, early (or highly proficient) bilinguals may have a less rigid boundary between their two languages and treat words from both languages as one integrated lexicon; therefore, they rely more on item-specific control. Most of the existing studies show a pattern consistent with this proposal, i.e., unbalanced bilinguals tend to exhibit switch cost asymmetry in language switching [3,6,8,13] (but see Reference [25]), whereas highly proficient bilinguals tend to show a reversed dominance effect [5,12]. For the highly proficient bilinguals, this pattern also seems to extend to their weaker L3 [5,18]. This suggests that it is the language control strategy employed by the bilinguals, rather than just the relative proficiency, that leads to one phenomenon or the other.

### 4.2. Alternatives to Inhibition in Bilingual Language Control

In designing the present study, we followed Green’s model of bilingual language control [2], where inhibition is assumed to be the central mechanism which prevents non-target language words from reaching speech output. Whilst this is the most influential view in the current literature, there are alternative proposals on how bilinguals might achieve appropriate language selection during speech production. One model suggests that lexical selection is language-specific, i.e., only words belonging to the target language are considered by the selection mechanism; therefore, there is no competition between languages [72,73]. This view is supported by findings from the picture–word interference paradigm, where picture naming is facilitated (rather than hindered) by a distractor word that is the translation-equivalent of the target name. Another model stipulates that language competition occurs at the semantic level. Specifically, the intended language is encoded in the preverbal message and this is sufficient to ensure higher activation levels of lexical nodes in that language [74,75].

In addition, there exists some evidence which seems incompatible with the inhibitory account of language control. For example, Runnqvist et al. [76] investigated the cumulative semantic interference (CSI) effect in a bilingual context. The monolingual version of this paradigm involves a picture-naming task where stimuli are chosen from a number of semantic categories. The CSI effect refers to the observation of longer naming latency for each additional picture named from the same semantic category. Such a pattern is assumed to reflect cumulative competition from the previously named objects in that category [77] (but see Reference [78]). Crucially, Runnqvist et al. showed that the slope of the CSI effect was unchanged even when objects belonging to the same semantic category were named in alternate languages. This speaks against Green’s inhibitory control model [2], which would predict an absence or reduced magnitude of the CSI effect, because inhibition during language alternation should cancel out (at least part of) the previous activation of the competitors. Such findings necessarily cast some doubts on the validity of language control via inhibition, especially on the whole-language scale.

Instead of substantial modifications to the inhibitory control model, Runnqvist et al. suggest that a simpler approach would be to consider the lexical access mechanism in bilinguals as qualitatively similar to that in monolinguals. In one of their proposed solutions, language membership serves as a semantic feature, which naturally passes down more activation to words belonging to the target language, thus resolving the competition between languages at the semantic level (see also References [74,75]). This proposal is compatible with the observations in the present study. Let us first consider the contrast between univalent and bivalent items. If language membership is encoded in the semantic representation, then the naming of each bivalent item in two different languages would be equivalent to naming two slightly different concepts. On the other hand, the naming of each univalent item would involve repeating the exact same concept. This may contribute to the valence cost. The asymmetry in the valence cost we observed was primarily driven by slower responses in L1 compared to L2 when naming bivalent items, and this could be explained by differential priming of the semantic representation when previously naming in the other language. If we assume that the L1 name is linked with more detailed semantic features while the L2 name is only linked to a subset of these [79], it then follows that the L2 name would receive more priming from the previous naming in L1 than vice versa, resulting in faster response speed in L2. In regard to how this proposal might accommodate the common observation of asymmetrical switch cost, La Heij [74] provided an explanation that the asymmetry may result from processes related to incorporating the language membership information into the preverbal message, rather than from inhibition.

### 4.3. Limitations

There are a few limitations in the present study, which may be informative for future research. Firstly, a possible limitation in the experimental design lies in the way univalent and bivalent items were presented. The target response for each univalent item appeared four times as much as the target response for each bivalent item in the experiment, because univalent items maintained the same item–language mappings, including when they appeared in the training block and when they appeared on filler trials in the testing block. As a result, the target response for univalent items would have received a lot more priming compared to the bivalent items. Such differential priming may be a contributing factor to any difference observed between the univalent and bivalent stimuli. For example, the fact that naming of univalent items was robustly faster and more accurate than bivalent items could be explained by the additional priming of the target response for the univalent items. We argued that, since the valence cost was asymmetrical between L1 and L2, this signified that item-specific inhibition was at least one component in the valence cost, even if other components also existed (see Section 2.3 for details). Therefore, the potential repetition priming effect does not compromise such interpretation of the valence cost. However, it would be better if this confound was removed altogether from the design. One possible approach is to reduce the number of bivalent items, so that the target label for each bivalent stimulus would be named an equal number of times as the target label for each univalent stimulus. While this leads to another potential concern—the bivalent stimuli themselves would be presented a lot more frequently than univalent stimuli—the latter is probably a less important concern than the differential priming on the target labels.

Secondly, a related point is that the small set of stimuli used in this study may not be a very good representation of a language. Since the stimuli were repeated many times in the experiment, participants’ responses (especially in the later part of the experiment) might have been driven more by learned associations between each picture stimulus and the motor plan(s) to name it, than lexical access. Specifically, for the univalent stimuli, participants always retrieved the same motor plan for each appearance of the same picture; for the bivalent stimuli, participants had to constantly reconfigure the mapping between the picture and the appropriate response according to the current language requirement. Therefore, it is possible that the item-specific inhibition was applied onto the stimulus–response bindings, and such inhibitory effects may reside in event files in episodic memory [80], rather than in the activation levels of lemmas. In addition, such a difference between univalent and bivalent stimuli, together with the possible repetition priming effect discussed above, may be responsible for the larger magnitude of the valence cost compared to the switch cost. As explained above, these issues should not compromise the interpretation of the asymmetry in the valence cost. However, the results may be more convincing if a larger set of picture stimuli were used.

Thirdly, the lack of strong evidence for switch cost and valence cost modulation by pre-SMA perturbation (Experiment 2) could be due to a number of technical factors; such results do not necessarily imply that the pre-SMA was not involved in whole-language or item-specific inhibition. One possible factor is that TMS might not have achieved the intended suppressive effect on pre-SMA. As most participants in this experiment had rather high motor threshold (mean 69%; range 60–76%), the calculated intensity to apply (48–61%) exceeded the maximum output available from the stimulator in the cTBS protocol (51%). Therefore, the stimulation applied on these participants could have been too weak to be effective. Moreover, previous studies showed that the suppressive effect of cTBS can be highly variable among individual participants [81,82]. Another possible factor is that behavioural measures such as naming latencies and accuracies may not be sensitive enough to detect the effect from the disruption of a brain region. For example, Pestalozzi et al. [83] applied excitatory and inhibitory TMS protocols to the dorsolateral prefrontal cortex in a language switching task; while TMS produced no visible behavioural effects, activity changes were detected in a number of brain regions in the electroencephalography (EEG) data. In our experiment, when the pre-SMA was perturbed, compensatory mechanisms might have been recruited to help mitigate the impact on participants’ behavioural performance. If this was the case, it would be very interesting to look into those compensatory mechanisms by recording the participants’ brain activity.

## 5. Conclusions

In this study, we examined a novel type of switching (“within-item switching”), alongside the commonly studied “trial-to-trial switching”, in a cued language switching paradigm. This design allowed us to capture the effect of whole-language and item-specific inhibition within the same experiment task and compare them side by side. Such comparison provided a unique opportunity to explore two levels of inhibitory control in language switching and their underlying neural mechanisms. Given the growing evidence on the involvement of the executive control brain network in bilingual speech production, we investigated whether a key brain region in this network, the pre-SMA, played a causal role in either level of language inhibition. Using non-invasive brain stimulation to disrupt the functioning of this brain region, we demonstrated a performance decrement in picture naming, consistent with its role in initiating speech in general. There was also indication of a possible role of the pre-SMA in whole-language and item-specific inhibition, although the evidence remains inconclusive and it awaits validation by future studies.

## Figures and Tables

**Table 1:** Characteristics of included participants in Experiment 1.

Characteristic	Mean	SD
Age	28.2	5.8
Age of first exposure to Mandarin	1.9	2.9
Age of first exposure to English	10.0	3.0
Mandarin MINT score ^a^	60.9	4.4
English MINT score ^a^	53.4	6.3
Mandarin listening ability ^b^	6.6	0.9
Mandarin speaking ability ^b^	6.4	0.9
Mandarin reading ability ^b^	6.7	0.8
Mandarin writing ability ^b^	6.5	0.9
English listening ability ^b^	5.5	0.9
English speaking ability ^b^	5.0	0.8
English reading ability ^b^	5.9	0.5
English writing ability ^b^	5.0	0.6
Percent Mandarin use currently ^c^	47.1	24.9
Percent English use currently ^c^	48.9	20.6
Percent Mandarin use during childhood ^c^	75.2	35.4
Percent English use during childhood ^c^	8.4	9.6
Switching frequency currently ^d^	4.2	1.2
Switching frequency in childhood ^d^	2.1	1.5

^a^ Maximum possible score in the Multilingual Naming Test (MINT) is 68 for each language. ^b^ Language proficiency based on self-ratings on a seven-point scale: 1 = little to no knowledge, 7 = like a native speaker. ^c^ Percentages for Mandarin and English use did not add up to exactly 100%, as some participants reported also speaking another variant of Chinese. ^d^ Switching frequency based on self-ratings on a six-point scale: 1 = never, 2 = very infrequently, 3 = occasionally, 4 = two to three times per conversation, 5 = several times per conversation, 6 = constantly.

**Table 2:** Mean reaction times (***left***) and error rates (***right***) in each condition in Experiment 1: “valence” (univalent vs. bivalent items) × “response language” (L1 vs. L2) × “transition type” (stay vs. switch trials).

	Univalent		Bivalent		Univalent		Bivalent	
	L1	L2	L1	L2	L1	L2	L1	L2
**Stay**	694	661	883	769	1.1%	1.7%	16.7%	11.7%
**Switch**	714	685	888	802	3.9%	1.7%	21.1%	13.9%

**Table 3:** Characteristics of included participants in Experiment 2.

Characteristic	Mean	SD
Age	24.6	4.9
Age of first exposure to Mandarin	1.5	2.3
Age of first exposure to English	8.6	3.8
Mandarin MINT score ^a^	60.8	4.5
English MINT score ^a^	47.4	8.4
Mandarin listening ability ^b^	6.8	0.7
Mandarin speaking ability ^b^	6.6	0.9
Mandarin reading ability ^b^	6.8	0.5
Mandarin writing ability ^b^	6.5	0.9
English listening ability ^b^	5.4	0.9
English speaking ability ^b^	4.9	1.1
English reading ability ^b^	5.4	0.9
English writing ability ^b^	4.9	1.0
Percent Mandarin use currently ^c^	61.2	21.9
Percent English use currently ^c^	35.4	17.1
Percent Mandarin use during childhood ^c^	81.9	22.9
Percent English use during childhood ^c^	10.6	11.0
Switching frequency currently ^d^	3.8	1.1
Switching frequency in childhood ^d^	2.0	1.1

^a^ Maximum possible score in the MINT test is 68 for each language. ^b^ Language proficiency based on self-ratings on a seven-point scale: 1 = little to no knowledge, 7 = like a native speaker. ^c^ Percentages for Mandarin and English use did not add up to exactly 100%, as some participants reported also speaking another variant of Chinese. ^d^ Switching frequency based on self-ratings on a six-point scale: 1 = never, 2 = very infrequently, 3 = occasionally, 4 = two to three times per conversation, 5 = several times per conversation, 6 = constantly.

**Table 4:** Mean reaction times (***top***) and error rates (***bottom***) in each condition in Experiment 2: “location of transcranial magnetic stimulation (TMS)” (control site vs. pre-SMA) × “valence” (univalent vs. bivalent items) × “response language” (L1 vs. L2) × “transition type” (stay vs. switch trials).

	**Control Site**				**Pre-SMA**			
	**Univalent**		**Bivalent**		**Univalent**		**Bivalent**	
	**L1**	**L2**	**L1**	**L2**	**L1**	**L2**	**L1**	**L2**
**Stay**	598	588	759	727	606	602	778	756
**Switch**	616	611	744	731	628	618	791	773
	**Control Site**				**Pre-SMA**			
	**Univalent**		**Bivalent**		**Univalent**		**Bivalent**	
	**L1**	**L2**	**L1**	**L2**	**L1**	**L2**	**L1**	**L2**
**Stay**	3.1%	1.0%	23.4%	24.0%	3.1%	0.5%	34.9%	17.7%
**Switch**	4.2%	3.1%	32.8%	26.0%	5.2%	2.1%	27.1%	26.0%

## Appendix A

Notes:

- When we talk about “univalent” and “bivalent” here, it is in regard to the context of this experiment. Technically, all of the stimuli are bivalent to the bilinguals, as they are able to name each picture in both languages. The definitions used here are consistent with Finkbeiner et al. [15].
- In the present experimental design, this is not guaranteed on all bivalent trials. However, it is true most of the time, as bivalent items are always named in the other language when they appear on filler trials, and there are twice as many fillers as critical trials in the testing block (see Section 2.1.3 for details on the experimental design and how the trial sequence is generated). If bivalent items are guaranteed to change language every time they appear on a critical trial, that should produce an even larger valence cost; the present design simply means that we are observing a smaller version of this effect.
- It would be better if we had a balanced number of Mandarin-dominant and English-dominant bilinguals; however, this was not achievable due to recruitment constraints.
- Note that the filler trials alone may be sufficient to create the bivalency, which means the training block may be redundant.
- The term “global” here means “both stay and switch trials”; it is not referring to “the whole language”.
- Note that the overall switch costs in these experiments were symmetrical. However, this does not prevent an interaction from showing up, if the switch cost pattern was different between univalent and bivalent stimuli. Therefore, the argument is still valid in this case.

## Appendix B

Statistical analysis results from Experiment 1.

**(1) Error analysis**

*> GLME model:*

Generalized linear mixed model fit by maximum likelihood (Laplace Approximation) [‘glmerMod’]
  Family: binomial (logit)
  Formula: error ~ valence * ttype * lang + (1 | subjectID) + (1 | item)
  Data: exp1_ER
  Control: glmerControl(optimizer = “bobyqa”)

AIC BIC logLik deviance df.resid
  783.6 836.4 −381.8 763.6 1430

Scaled residuals:
  Min 1Q Median 3Q Max
  −0.5900 −0.4005 −0.1919 −0.1194 9.3559

Random effects:
  Groups Name Variance Std.Dev.
  subjectID (Intercept) 0.03475 0.1864
  item (Intercept) 0.03544 0.1883
  Number of obs: 1440, groups: subjectID, 15; item, 8

Fixed effects:
  Estimate Std. Error z value Pr(>|z|)
  (Intercept) −4.5141 0.7147 −6.316 0.000000000268 ***
  valenceBi 2.8853 0.7379 3.910 0.000092344847 ***
  ttypeSwitch 1.2831 0.8061 1.592 0.111
  langL2 0.3960 0.9171 0.432 0.666
  valenceBi:ttypeSwitch −0.9888 0.8506 −1.162 0.245
  valenceBi:langL2 −0.8217 0.9680 −0.849 0.396
  ttypeSwitch:langL2 −1.2831 1.1501 −1.116 0.265
  valenceBi:ttypeSwitch:langL2 1.1901 1.2234 0.973 0.331
  ---
  Signif. codes: 0 ‘***’ 0.001 ‘**’ 0.01 ‘*’ 0.05 ‘.’ 0.1 ‘ ‘ 1

Correlation of Fixed Effects:
  (Intr) valncB ttypSw langL2 vlnB:S vlB:L2 ttS:L2
  valenceBi −0.955
  ttypeSwitch −0.871 0.844
  langL2 −0.767 0.744 0.679
  vlncB:ttypS 0.825 −0.863 −0.948 −0.643
  vlncB:lngL2 0.728 −0.763 −0.643 −0.947 0.658
  ttypSwtc:L2 0.610 −0.591 −0.701 −0.795 0.664 0.753
  vlncB:tS:L2 −0.574 0.600 0.658 0.747 −0.695 −0.787 −0.940

Analysis of Deviance Table (Type II Wald chisquare tests)

Response: error
  Chisq Df Pr(>Chisq)
  valence 57.4183 1 3.523e-14 ***
  ttype 2.3911 1 0.12203
  lang 5.5610 1 0.01836 *
  valence:ttype 0.4577 1 0.49870
  valence:lang 0.0182 1 0.89280
  ttype:lang 0.3479 1 0.55532
  valence:ttype:lang 0.9463 1 0.33067
  ---
  Signif. codes: 0 ‘***’ 0.001 ‘**’ 0.01 ‘*’ 0.05 ‘.’ 0.1 ‘ ‘ 1

**(2) Reaction time analysis**

*> LME model (raw RT):*

Linear mixed model fit by maximum likelihood. t-tests use Satterthwaite’s method [‘lmerModLmerTest’]
  Formula: RT ~ valence * ttype * lang + (1 | subjectID) + (1 | item)
  Data: exp1_RT
  Control: lmerControl(optimizer = “bobyqa”)

AIC BIC logLik deviance df.resid
  17096.5 17153.4 −8537.3 17074.5 1290

Scaled residuals:
  Min 1Q Median 3Q Max
  −2.4484 −0.6075 −0.1290 0.3592 7.0417

Random effects:
  Groups Name Variance Std.Dev.
  subjectID (Intercept) 11283 106.22
  item (Intercept) 2317 48.13
  Residual 27729 166.52
  Number of obs: 1301, groups: subjectID, 15; item, 8

Fixed effects:
  Estimate Std. Error df t value Pr(>|t|)
  (Intercept) 694.313 34.700 26.750 20.009 <2e-16 ***
  valenceBi 167.596 18.981 1285.279 8.830 <2e-16 ***
  ttypeSwitch 17.193 17.808 1278.921 0.965 0.3345
  langL2 −25.596 18.109 1285.085 −1.413 0.1578
  valenceBi:ttypeSwitch 5.280 26.558 1279.129 0.199 0.8425
  valenceBi:langL2 −61.948 26.511 1284.274 −2.337 0.0196 *
  ttypeSwitch:langL2 4.824 25.128 1278.894 0.192 0.8478
  valenceBi:ttypeSwitch:langL2 7.444 37.108 1279.087 0.201 0.8410
  ---
  Signif. codes: 0 ‘***’ 0.001 ‘**’ 0.01 ‘*’ 0.05 ‘.’ 0.1 ‘ ’ 1

Correlation of Fixed Effects:
  (Intr) valncB ttypSw langL2 vlnB:S vlB:L2 ttS:L2
  valenceBi −0.250
  ttypeSwitch −0.253 0.462
  langL2 −0.262 0.481 0.485
  vlncB:ttypS 0.169 −0.682 −0.671 −0.322
  vlncB:lngL2 0.180 −0.718 −0.330 −0.682 0.487
  ttypSwtc:L2 0.180 −0.328 −0.709 −0.690 0.475 0.471
  vlncB:tS:L2 −0.121 0.487 0.480 0.464 −0.716 −0.688 −0.677
  Analysis of Deviance Table (Type II Wald chisquare tests)

Response: RT
  Chisq Df Pr(>Chisq)
  valence 215.7919 1 < 2.2e-16 ***
  ttype 6.6664 1 0.009825 **
  lang 27.7909 1 1.352e-07 ***
  valence:ttype 0.2403 1 0.623982
  valence:lang 9.1807 1 0.002446 **
  ttype:lang 0.1985 1 0.655922
  valence:ttype:lang 0.0402 1 0.841017
  ---
  Signif. codes: 0 ‘***’ 0.001 ‘**’ 0.01 ‘*’ 0.05 ‘.’ 0.1 ‘ ’ 1

*> LME model (log-transformed RT):*

Linear mixed model fit by maximum likelihood. t-tests use Satterthwaite’s method [‘lmerModLmerTest’]
  Formula: log(RT) ~ valence * ttype * lang + (1 | subjectID) + (1 | item)
  Data: exp1_RT
  Control: lmerControl(optimizer = “bobyqa”)

AIC BIC logLik deviance df.resid
  -503.1 −446.2 262.5 −525.1 1290

Scaled residuals:
  Min 1Q Median 3Q Max
  −3.4618 −0.6601 −0.0990 0.4947 4.6041

Random effects:
  Groups Name Variance Std.Dev.
  subjectID (Intercept) 0.018271 0.13517
  item (Intercept) 0.003546 0.05955
  Residual 0.036868 0.19201
  Number of obs: 1301, groups: subjectID, 15; item, 8

Fixed effects:
  Estimate Std. Error df t value Pr(>|t|)
  (Intercept) 6.512e+00 4.333e-02 2.581e+01 150.295 <2e-16 ***
  valenceBi 2.070e-01 2.189e-02 1.285e+03 9.458 <2e-16 ***
  ttypeSwitch 2.749e-02 2.053e-02 1.279e+03 1.339 0.1808
  langL2 −2.767e-02 2.089e-02 1.285e+03 −1.325 0.1855
  valenceBi:ttypeSwitch -7.768e-03 3.062e-02 1.279e+03 −0.254 0.7998
  valenceBi:langL2 -6.820e-02 3.057e-02 1.284e+03 −2.231 0.0259 *
  ttypeSwitch:langL2 9.590e-04 2.897e-02 1.279e+03 0.033 0.9736
  valenceBi:ttypeSwitch:langL2 2.274e-02 4.279e-02 1.279e+03 0.531 0.5952
  ---
  Signif. codes: 0 ‘***’ 0.001 ‘**’ 0.01 ‘*’ 0.05 ‘.’ 0.1 ‘ ’ 1

Correlation of Fixed Effects:
  (Intr) valncB ttypSw langL2 vlnB:S vlB:L2 ttS:L2
  valenceBi −0.231
  ttypeSwitch −0.234 0.462
  langL2 −0.242 0.481 0.485
  vlncB:ttypS 0.156 −0.682 −0.671 −0.322
  vlncB:lngL2 0.166 −0.718 −0.330 −0.682 0.487
  ttypSwtc:L2 0.166 −0.327 −0.709 −0.690 0.475 0.471
  vlncB:tS:L2 −0.111 0.487 0.480 0.464 −0.716 −0.688 −0.677

Analysis of Deviance Table (Type II Wald chisquare tests)

Response: log(RT)
  Chisq Df Pr(>Chisq)
  valence 250.0791 1 < 2.2e-16 ***
  ttype 7.8490 1 0.005085 **
  lang 23.7926 1 1.073e-06 ***
  valence:ttype 0.0329 1 0.856028
  valence:lang 6.6031 1 0.010180 *
  ttype:lang 0.2854 1 0.593208
  valence:ttype:lang 0.2825 1 0.595092
  ---
  Signif. codes: 0 ‘***’ 0.001 ‘**’ 0.01 ‘*’ 0.05 ‘.’ 0.1 ‘ ’ 1

*> Permutation test on LME model:*

Analysis of Variance Table of type I with Kenward-Roger approximation for degrees of freedom
  Sum Sq Mean Sq NumDF DenDF F value Pr(>F) DDf p.value Perm.p
  valence 5943550 5943550 1 1278.4 213.1799 0.00000 1278.4 0.00000 0.001
  ttype 177390 177390 1 1272.2 6.3625 0.01178 1272.2 0.01178 0.012
  lang 755004 755004 1 1279.0 27.0800 0.00000 1279.0 0.00000 0.002
  valence:ttype 6140 6140 1 1272.2 0.2202 0.63896 1272.2 0.63896 0.623
  valence:lang 253992 253992 1 1279.1 9.1101 0.00259 1279.1 0.00259 0.008
  ttype:lang 5494 5494 1 1272.1 0.1971 0.65718 1272.1 0.65718 0.656
  valence:ttype:lang 1105 1105 1 1272.2 0.0397 0.84220 1272.2 0.84220 0.846

*> Follow-up tests to unpack the interaction between “valence” and “language”:*

lang = L1:
  valence emmean SE df lower.CL upper.CL
  Uni 703 34.4 24.8 632 774
  Bi 873 34.6 25.5 802 944

lang = L2:
  valence emmean SE df lower.CL upper.CL
  Uni 680 34.3 24.6 609 750
  Bi 792 34.5 25.1 721 863

Results are averaged over the levels of: ttype
  Degrees-of-freedom method: kenward-roger
  Confidence level used: 0.95

contrast lang estimate SE df t.ratio p.value
  Uni - Bi L1 −170 14.0 1294 −12.203 <0.0001
  Uni - Bi L2 −112 13.2 1291 −8.477 <0.0001

Results are averaged over the levels of: ttype
  Degrees-of-freedom method: kenward-roger
  P value adjustment: bonferroni method for 2 tests

## Appendix C

Statistical analysis results from Experiment 2.

**(1) Error analysis**

*> GLME model:*

Generalized linear mixed model fit by maximum likelihood (Laplace Approximation) [‘glmerMod’]
  Family: binomial (logit)
  Formula: error ~ tms * valence * ttype * lang + (1 | subjectID) + (1 | item)
  Data: exp2_ER
  Control: glmerControl(optimizer = “bobyqa”)

AIC BIC logLik deviance df.resid
  2000.0 2108.6 −982.0 1964.0 3054

Scaled residuals:
  Min 1Q Median 3Q Max
  −1.4616 −0.3953 −0.1882 −0.0821 12.4798

Random effects:
  Groups Name Variance Std.Dev.
  subjectID (Intercept) 0.76039 0.872
  item (Intercept) 0.04409 0.210
  Number of obs: 3072, groups: subjectID, 16; item, 8

Fixed effects:
  Estimate Std. Error z value Pr(>|z|)
  (Intercept) −3.800e+00 4.795e-01 −7.925 2.27e-15 ***
  tmsps 2.915e-05 5.896e-01 0.000 1.000
  valenceBi 2.423e+00 4.563e-01 5.310 1.10e-07 ***
  ttypeSwitch 3.060e-01 5.538e-01 0.553 0.581
  langL2 −1.089e+00 8.234e-01 −1.322 0.186
  tmsps:valenceBi 6.446e-01 6.379e-01 1.010 0.312
  tmsps:ttypeSwitch 2.415e-01 7.671e-01 0.315 0.753
  valenceBi:ttypeSwitch 2.302e-01 6.054e-01 0.380 0.704
  tmsps:langL2 -7.019e-01 1.358e+00 −0.517 0.605
  valenceBi:langL2 1.127e+00 8.625e-01 1.307 0.191
  ttypeSwitch:langL2 8.278e-01 9.908e-01 0.835 0.403
  tmsps:valenceBi:ttypeSwitch −1.202e+00 8.397e-01 −1.432 0.152
  tmsps:valenceBi:langL2 −3.696e-01 1.405e+00 −0.263 0.793
  tmsps:ttypeSwitch:langL2 3.726e-02 1.585e+00 0.024 0.981
  valenceBi:ttypeSwitch:langL2 −1.238e+00 1.051e+00 −1.178 0.239
  tmsps:valenceBi:ttypeSwitch:langL2 1.350e+00 1.662e+00 0.813 0.416
  ---
  Signif. codes: 0 ‘***’ 0.001 ‘**’ 0.01 ‘*’ 0.05 ‘.’ 0.1 ‘ ’ 1

Analysis of Deviance Table (Type II Wald chisquare tests)

Response: error
  Chisq Df Pr(>Chisq)
  tms 0.0153 1 0.901709
  valence 233.9853 1 < 2.2e-16 ***
  ttype 3.9657 1 0.046436 *
  lang 10.5175 1 0.001182 **
  tms:valence 0.0666 1 0.796315
  tms:ttype 1.0820 1 0.298242
  valence:ttype 2.3255 1 0.127266
  tms:lang 2.6250 1 0.105194
  valence:lang 2.0218 1 0.155055
  ttype:lang 1.8891 1 0.169304
  tms:valence:ttype 1.4000 1 0.236731
  tms:valence:lang 0.6289 1 0.427750
  tms:ttype:lang 7.0611 1 0.007877 **
  valence:ttype:lang 0.7354 1 0.391145
  tms:valence:ttype:lang 0.6603 1 0.416458
  ---
  Signif. codes: 0 ‘***’ 0.001 ‘**’ 0.01 ‘*’ 0.05 ‘.’ 0.1 ‘ ’ 1

*> Follow-up tests to unpack the 3-way interaction between “TMS location”, “transition type” and “language”:*

tms = vt:
  ttype lang emmean SE df asymp.LCL asymp.UCL
  Stay L1 −2.59 0.326 Inf −3.23 −1.95
  Switch L1 −2.17 0.307 Inf −2.77 −1.57
  Stay L2 −3.11 0.434 Inf −3.96 −2.26
  Switch L2 −2.48 0.325 Inf −3.12 −1.85

tms = ps:
  ttype lang emmean SE df asymp.LCL asymp.UCL
  Stay L1 −2.27 0.323 Inf −2.90 −1.63
  Switch L1 −2.20 0.299 Inf −2.79 −1.62
  Stay L2 −3.68 0.560 Inf −4.78 −2.58
  Switch L2 −2.70 0.355 Inf −3.39 −2.00

Results are averaged over the levels of: valence
  Results are given on the logit (not the response) scale.
  Confidence level used: 0.95

tms = vt:
  ttype_pairwise lang_pairwise estimate SE df z.ratio p.value
  Stay - Switch L1 - L2 0.209 0.526 Inf 0.398 0.6909

tms = ps:
  ttype_pairwise lang_pairwise estimate SE df z.ratio p.value
  Stay - Switch L1 - L2 0.921 0.644 Inf 1.430 0.1528

Results are averaged over the levels of: valence
  Note: contrasts are still on the log.o.r. scale

**(2) Reaction time analysis**

*> LME model (raw RT):*

Linear mixed model fit by maximum likelihood. t-tests use Satterthwaite’s method [‘lmerModLmerTest’]
  Formula: RT ~ tms * valence * ttype * lang + (1 | subjectID) + (1 | item)
  Data: exp2_RT
  Control: lmerControl(optimizer = “bobyqa”)

AIC BIC logLik deviance df.resid
  33315.5 33426.7 −16638.8 33277.5 2552

Scaled residuals:
  Min 1Q Median 3Q Max
  −2.4767 −0.5420 −0.1235 0.3356 11.7271

Random effects:
  Groups Name Variance Std.Dev.
  subjectID (Intercept) 11378 106.7
  item (Intercept) 1989 44.6
  Residual 23606 153.6
  Number of obs: 2571, groups: subjectID, 16; item, 8

Fixed effects:
  Estimate Std. Error df t value Pr(>|t|)
  (Intercept) 597.411 33.052 28.332 18.075 <2e-16 ***
  tmsps 7.523 16.089 2548.011 0.468 0.6401
  valenceBi 164.865 17.256 2550.882 9.554 <2e-16 ***
  ttypeSwitch 18.727 16.222 2548.011 1.154 0.2484
  langL2 −9.721 16.203 2551.531 −0.600 0.5486
  tmsps:valenceBi 8.385 24.772 2548.249 0.338 0.7350
  tmsps:ttypeSwitch 1.678 22.864 2548.017 0.073 0.9415
  valenceBi:ttypeSwitch −29.910 24.810 2548.334 −1.206 0.2281
  tmsps:langL2 6.442 22.647 2548.025 0.284 0.7761
  valenceBi:langL2 −40.855 24.361 2549.373 −1.677 0.0937
  ttypeSwitch:langL2 2.821 22.724 2548.014 0.124 0.9012
  tmsps:valenceBi:ttypeSwitch 21.333 35.192 2548.380 0.606 0.5444
  tmsps:valenceBi:langL2 7.767 34.498 2548.162 0.225 0.8219
  tmsps:ttypeSwitch:langL2 −8.202 32.103 2548.020 −0.255 0.7984
  valenceBi:ttypeSwitch:langL2 17.303 34.821 2548.220 0.497 0.6193
  tmsps:valenceBi:ttypeSwitch:langL2 1.117 49.088 2548.213 0.023 0.9818
  ---
  Signif. codes: 0 ‘***’ 0.001 ‘**’ 0.01 ‘*’ 0.05 ‘.’ 0.1 ‘ ’ 1

Analysis of Deviance Table (Type II Wald chisquare tests)

Response: RT
  Chisq Df Pr(>Chisq)
  tms 10.2989 1 0.001331 **
  valence 516.1176 1 < 2.2e-16 ***
  ttype 5.8391 1 0.015674 *
  lang 9.4246 1 0.002141 **
  tms:valence 3.5719 1 0.058767.
  tms:ttype 0.3183 1 0.572615
  valence:ttype 0.6636 1 0.415286
  tms:lang 0.2391 1 0.624838
  valence:lang 5.1166 1 0.023698 *
  ttype:lang 0.2729 1 0.601392
  tms:valence:ttype 0.7972 1 0.371944
  tms:valence:lang 0.1149 1 0.734590
  tms:ttype:lang 0.1011 1 0.750459
  valence:ttype:lang 0.5298 1 0.466709
  tms:valence:ttype:lang 0.0005 1 0.981838
  ---
  Signif. codes: 0 ‘***’ 0.001 ‘**’ 0.01 ‘*’ 0.05 ‘.’ 0.1 ‘ ’ 1

*> LME model (log-transformed RT):*

Linear mixed model fit by maximum likelihood. t-tests use Satterthwaite’s method [‘lmerModLmerTest’]
  Formula: log(RT) ~ tms * valence * ttype * lang + (1 | subjectID) + (1 | item)
  Data: exp2_RT
  Control: lmerControl(optimizer = “bobyqa”)

AIC BIC logLik deviance df.resid
  −1341.3 −1230.1 689.6 −1379.3 2552

Scaled residuals:
  Min 1Q Median 3Q Max
  −2.7452 −0.6402 −0.1076 0.4877 6.0468

Random effects:
  Groups Name Variance Std.Dev.
  subjectID (Intercept) 0.020628 0.14363
  item (Intercept) 0.003841 0.06197
  Residual 0.032939 0.18149
  Number of obs: 2571, groups: subjectID, 16; item, 8

Fixed effects:
  Estimate Std. Error df t value Pr(>|t|)
  (Intercept) 6.370e+00 4.421e-02 2.705e+01 144.096 <2e-16 ***
  tmsps 5.451e-03 1.901e-02 2.548e+03 0.287 0.774
  valenceBi 2.251e-01 2.039e-02 2.550e+03 11.041 <2e-16 ***
  ttypeSwitch 2.588e-02 1.916e-02 2.548e+03 1.351 0.177
  langL2 −1.014e-02 1.914e-02 2.551e+03 −0.530 0.596
  tmsps:valenceBi 7.237e-03 2.926e-02 2.548e+03 0.247 0.805
  tmsps:ttypeSwitch 1.237e-02 2.701e-02 2.548e+03 0.458 0.647
  valenceBi:ttypeSwitch −3.082e-02 2.931e-02 2.548e+03 −1.052 0.293
  tmsps:langL2 1.361e-02 2.675e-02 2.548e+03 0.509 0.611
  valenceBi:langL2 −5.912e-02 2.878e-02 2.549e+03 −2.054 0.040 *
  ttypeSwitch:langL2 6.306e-03 2.684e-02 2.548e+03 0.235 0.814
  tmsps:valenceBi:ttypeSwitch 1.320e-02 4.157e-02 2.548e+03 0.318 0.751
  tmsps:valenceBi:langL2 8.756e-03 4.075e-02 2.548e+03 0.215 0.830
  tmsps:ttypeSwitch:langL2 −1.903e-02 3.792e-02 2.548e+03 −0.502 0.616
  valenceBi:ttypeSwitch:langL2 2.135e-02 4.113e-02 2.548e+03 0.519 0.604
  tmsps:valenceBi:ttypeSwitch:langL2 −2.654e-03 5.799e-02 2.548e+03 −0.046 0.963
  ---
  Signif. codes: 0 ‘***’ 0.001 ‘**’ 0.01 ‘*’ 0.05 ‘.’ 0.1 ‘ ’ 1

Analysis of Deviance Table (Type II Wald chisquare tests)

Response: log(RT)
  Chisq Df Pr(>Chisq)
  tms 8.7796 1 0.0030462 **
  valence 657.6509 1 < 2.2e-16 ***
  ttype 11.6650 1 0.0006369 ***
  lang 10.7716 1 0.0010307 **
  tms:valence 1.4715 1 0.2251123
  tms:ttype 0.2868 1 0.5922502
  valence:ttype 0.9213 1 0.3371374
  tms:lang 0.2645 1 0.6070647
  valence:lang 9.3186 1 0.0022684 **
  ttype:lang 0.1369 1 0.7114306
  tms:valence:ttype 0.1669 1 0.6829159
  tms:valence:lang 0.0660 1 0.7972995
  tms:ttype:lang 0.4938 1 0.4822207
  valence:ttype:lang 0.4766 1 0.4899545
  tms:valence:ttype:lang 0.0021 1 0.9634929
  ---
  Signif. codes: 0 ‘***’ 0.001 ‘**’ 0.01 ‘*’ 0.05 ‘.’ 0.1 ‘ ’ 1

*> Permutation test on LME model:*

Analysis of Variance Table of type I with Kenward-Roger approximation for degrees of freedom
  Sum Sq Mean Sq NumDF DenDF F value Pr(>F) DDf p.value Perm.p
  tms 247944 247944 1 2533.2 10.4417 0.00125 2533.2 0.00125 0.061
  valence 12144022 12144022 1 2540.8 511.4220 0.00000 2540.8 0.00000 0.001
  ttype 141298 141298 1 2533.1 5.9505 0.01478 2533.1 0.01478 0.022
  lang 217356 217356 1 2540.0 9.1535 0.00251 2540.0 0.00251 0.118
  tms:valence 78429 78429 1 2533.2 3.3029 0.06928 2533.2 0.06928 0.079
  tms:ttype 9369 9369 1 2533.2 0.3946 0.52997 2533.2 0.52997 0.568
  valence:ttype 16069 16069 1 2533.1 0.6767 0.41080 2533.1 0.41080 0.422
  tms:lang 5719 5719 1 2533.2 0.2408 0.62365 2533.2 0.62365 0.619
  valence:lang 123969 123969 1 2537.3 5.2207 0.02240 2537.3 0.02240 0.025
  ttype:lang 6931 6931 1 2533.1 0.2919 0.58906 2533.1 0.58906 0.573
  tms:valence:ttype 19009 19009 1 2533.2 0.8005 0.37102 2533.2 0.37102 0.378
  tms:valence:lang 2922 2922 1 2533.2 0.1230 0.72579 2533.2 0.72579 0.728
  tms:ttype:lang 2347 2347 1 2533.1 0.0988 0.75328 2533.1 0.75328 0.756
  valence:ttype:lang 12511 12511 1 2533.2 0.5269 0.46799 2533.2 0.46799 0.470
  tms:valence:ttype:lang 12 12 1 2533.2 0.0005 0.98174 2533.2 0.98174 0.980

*> Follow-up tests to unpack the interaction between “valence” and “language”:*

lang = L1:
  valence emmean SE df lower.CL upper.CL
  Uni 611 32.2 24.5 544 677
  Bi 770 32.4 25.2 704 837

lang = L2:
  valence emmean SE df lower.CL upper.CL
  Uni 604 32.2 24.5 537 670
  Bi 735 32.4 25.0 669 802

Results are averaged over the levels of: tms, ttype
  Degrees-of-freedom method: kenward-roger
  Confidence level used: 0.95

contrast lang estimate SE df t.ratio p.value
  Uni - Bi L1 −159 9.08 2570 −17.558 <0.0001
  Uni - Bi L2 −131 8.83 2570 −14.878 <0.0001

Results are averaged over the levels of: tms, ttype
  Degrees-of-freedom method: kenward-roger
  P value adjustment: bonferroni method for 2 tests

*> Follow-up tests to unpack the marginal interaction between “TMS location” and “valence”:*

tms = vt:
  valence emmean SE df lower.CL upper.CL
  Uni 603 32.2 24.5 536 669
  Bi 736 32.4 25.1 670 803

tms = ps:
  valence emmean SE df lower.CL upper.CL
  Uni 612 32.2 24.5 546 679
  Bi 769 32.4 25.0 702 836

Results are averaged over the levels of: ttype, lang
  Degrees-of-freedom method: kenward-roger
  Confidence level used: 0.95

contrast tms estimate SE df t.ratio p.value
  Uni - Bi vt -134 8.95 2570 -14.952 <0.0001
  Uni - Bi ps -157 8.85 2569 -17.740 <0.0001

Results are averaged over the levels of: ttype, lang
  Degrees-of-freedom method: kenward-roger
  P value adjustment: bonferroni method for 2 tests
